# Semiconducting Polymer Nanoparticles Enable Light‐Controlled Bidirectional Modulation of Nitric Oxide in Endothelial Cells

**DOI:** 10.1002/advs.202522894

**Published:** 2026-02-20

**Authors:** Camilla Marzuoli, Andrea Pianetti, Elena Mancinelli, Anthea Villano, Matteo Vailati, Paola Lagonegro, Miryam Criado‐Gonzalez, Montserrat Climent, Hansel Comas‐Rojas, Leonardo Elia, Francesco Moccia, Gabriele Tullii, Maria Rosa Antognazza

**Affiliations:** ^1^ Center for Nano Science and Technology Istituto Italiano di Tecnologia Milano Italy; ^2^ Institute of Polymer Science and Technology (ICTP‐CSIC) Madrid Spain; ^3^ Department of Biomedical Sciences Humanitas University Pieve Emanuele Italy; ^4^ Humanitas Cardio Center IRCCS Humanitas Research Hospital Rozzano Italy; ^5^ Department of molecular and translational medicine University of Brescia Brescia Italy; ^6^ Department of Medicine and Health Sciences “V. Tiberio” University of Molise Campobasso Italy

**Keywords:** conjugated polymer, endothelial cells, light, nitric oxide, organic bioelectronics, photo‐redox modulation, semiconducting polymer nanoparticles

## Abstract

Endothelial‐derived nitric oxide (·NO) is a key signaling molecule in the vascular system, exerting concentration‐dependent control over critical cellular functions such as angiogenesis, vascular tone, and endothelial barrier integrity. Tools for achieving reversible, spatiotemporally resolved modulation of intracellular ·NO, without pharmacological or genetic manipulation, are currently lacking. Here, we present a light‐activated, nanoparticle (NP)‐based strategy enabling bidirectional control of endogenous ·NO in endothelial cells. Composite NPs, based on poly(3‐hexylthiophene), P3HT, and poly(3,4‐ethylene‐dioxythiophene):poly(styrenesulfonate), PEDOT:PSS polymers, are efficiently internalized in human (HUVEC) and murine (H5V) endothelial cells. In dark, NP uptake induces a ROS‐dependent, intracellular ·NO increase (+50% and +100% in HUVEC and H5V, respectively, vs. controls), a metabolic shift toward glycolysis and upregulation of both endothelial nitric oxide synthase (eNOS, +50%) and induced nitric oxide synthase (iNOS, +40%). NP photostimulation reverses this response, decreasing ·NO below basal levels, up to −40% in HUVEC and H5V, via ROS‐mediated scavenging and iNOS downregulation (−40%), partially restoring oxidative phosphorylation metabolism. Importantly, the photoexcitation protocol is compatible with perspective in vivo use, in terms of source type (LEDs) and power density (6 mW/cm^2^). Our approach represents an innovative strategy for bidirectional endothelial ·NO modulation, providing new opportunities in the emerging field of photo‐redox medicine.

## Graphical abstract

Bimodal modulation of nitric oxide in endothelial cells is achieved by light‐sensitive polymer nanoparticles. In dark, P3HT/PEDOT:PSS NPs boost intracellular ·NO, upregulate both endothelial and induced nitric oxide synthase, and drive a metabolic shift toward glycolysis. Upon photoexcitation, the same NPs suppress ·NO below basal level, down‐regulate iNOS and restore oxidative phosphorylation.

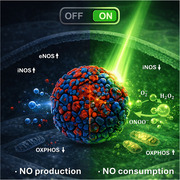

## 1 Introduction

Nitric oxide (·NO) is a gaseous signaling molecule involved in the regulation of numerous physiological processes across the nervous, immune, gastrointestinal and cardiovascular systems [1].

Within the vasculature, ·NO plays a fundamental role in regulating vasodilation and smooth muscle relaxation, maintaining blood pressure and flow, and modulating endothelial barrier integrity [1, 2]. In addition, ·NO is essential for maintaining vascular homeostasis by stimulating angiogenesis and vasculogenesis, preventing platelet aggregation and vascular smooth muscle cell proliferation, and regulating endothelial‐immune cell interactions [2, 3]. Endothelial cells, which line the inner walls of blood vessels, are the primary source of vascular ·NO, synthesizing it through various isoforms of Nitric Oxide Synthase (NOS), particularly through the activity of endothelial NOS (eNOS), which mediates ·NO production under physiological conditions, and inducible NOS (iNOS), which is typically upregulated in response to inflammatory stimuli [4]. In brief, endothelial‐derived ·NO is a critical gasotransmitter for the maintenance of cardiovascular homeostasis [1, 5, 6]. Endothelial dysfunction, which is a hallmark of cardiovascular disorders, vascular dementia and neurodegenerative disorders, is associated with a severe impairment of ·NO bioavailability, leading to excessive vascular permeability, vascular inflammation, enhanced vasoconstriction and hypertension. Therefore, enhancing endogenous ·NO production is regarded as one of the most suitable therapeutic strategies to restore endothelial signaling and reduce cardiometabolic risk [1, 7].

A defining feature of ·NO as a signaling mediator is its bimodal nature, whereby its cellular effects are highly dependent on local concentration. At physiological levels, ·NO supports homeostasis and vascular health, while at elevated concentrations, it can contribute to oxidative stress and to the onset of pathological processes [1]. This concentration‐dependent characteristics highlights the need for tools that allow precise and reversible modulation of intracellular ·NO levels, a key goal in redox medicine and vascular therapeutics [8].

Current pharmacological interventions aimed at regulating ·NO levels involve either ·NO increase, by delivering ·NO donors or drugs able to enhance ·NO synthesis [1, 7], or ·NO depletion, by using ·NO scavengers and inhibitors of ·NO synthases (NOS) [9, 10]. Importantly, the management of pharmacological ·NO‐related therapies must account for potential side effects, mainly due to the lack of spatial and temporal resolution of administration and of reversibility [7, 11]. A spatiotemporally controlled and reversible modulation of ·NO is therefore highly desirable. In this scenario, the use of light as a modulation trigger offers unique advantages, combining non‐invasive, highly tunable control with excellent temporal and spatial precision [12]. Photobiomodulation (PBM) exploits specific light wavelengths to stimulate endogenous NOS activity, enabling temporally controlled and low‐toxicity modulation of ·NO signaling [13]. However, PBM typically induces only modest changes in ·NO levels, with efficacy restricted to narrow wavelength ranges (e.g., 620–680 and 780–850 nm), and provides limited spatial resolution at the subcellular level [13, 14].

To overcome these limitations, the integration of photoresponsive nanomaterials offers a promising strategy [15, 16]. Such materials can act as phototransducers, converting light energy into chemical signals – including reactive oxygen species (ROS) – to trigger or suppress intracellular pathways. By tuning their optical and surface properties, these nanoplatforms can be customized for different therapeutic contexts.

To date, most nanomaterial‐based strategies for ·NO control focus on the release of exogenous ·NO, relying on the photochemical activation of ·NO donors, such as photoresponsive molecules, crystals, or nanoparticles (NPs), that release ·NO upon light stimulation [17, 18, 19]. Although these methods provide excellent spatiotemporal precision, they are inherently unidirectional and lack reversibility, offering limited control over intracellular ·NO levels. This limitation is particularly critical given the dichotomous nature of ·NO signaling, which requires the ability to finely tune ·NO levels in both directions, enabling the possibility to suppress or reverse the increase in NO.

Moreover, direct ·NO delivery relies on exogenous administration, bypassing the intrinsic regulatory mechanisms of the cell and potentially disrupting homeostasis. In contrast, the possibility to stimulate endogenous ·NO production represents a more physiological approach, enabling targeted modulation of intracellular pathways while minimizing the risk of overdosing and oxidative stress.

To overcome these challenges, we introduce a novel strategy that combines material‐mediated stimulation of endogenous ·NO production with light‐driven modulation, enabling a bidirectional, reversible control of endogenous ·NO levels, using a single material interface. In this context, the use of a material that remains inert in the absence of light but becomes photoactive upon illumination represents an optimal strategy to enable bidirectional ·NO modulation by decoupling the intrinsic properties of the material from its light‐induced effects. Among other possibilities, organic semiconductors, and conjugated polymers in particular, stand out as prosing candidates due to their favourable optoelectronic properties, excellent biocompatibility and processability easiness [20, 21, 22, 23, 24]. Several recent works demonstrated that poly(3‐hexylthiophene) (P3HT), a conjugated polymer widely studied in organic bioelectronics [25, 26, 27], exhibit stable and efficient photocatalytic activity in aqueous, biological‐like environment, either in the form of thin film or as beads in colloidal dispersion [28, 29, 30, 31, 32, 33, 34, 35]. P3HT photoelectrochemical activity was successfully employed to modulate cellular activity by efficiently converting light stimuli into cellular biochemical responses [28, 29, 31, 36, 37]. In particular, P3HT NPs were reported to reliably regulate intracellular ROS levels, in a fully safe concentration range and preserving *eu*stress condition, that is, a physiological range of oxidative stress supporting cellular adaptation and function [30, 31, 38]. Intracellular ROS act as key secondary messengers involved in various signaling pathways, including those that regulate vascular function and endothelial homeostasis [37, 39, 40]. Importantly to this work, we demonstrated that composite P3HT‐based NPs enable a light‐controlled, bimodal regulation of angiogenic processes, underscoring their potential as a versatile platform for therapeutic applications in the endothelium [31].

The interplay among ROS and other key mediators, such as ·NO, within the reactive species interactome has been the subject of extensive recent research in fundamental biology, and unidirectional, ·NO optical modulation has been also recently demonstrated by a variety of functional materials, mostly through catalytic or photothermal processes [41, 42, 43]. In contrast, there are no reports, to the best of our knowledge, about the use of conjugated semiconducting polymers to optically trigger ·NO release in a spatio‐temporally precise, bimodal and reversible way.

In this study, we not only leverage on the capability of P3HT‐based NPs to efficiently modulate ROS and angiogenesis in a bimodal manner in endothelial cells [31], but we also investigate their potential for reversible, optically controlled ·NO regulation. Using fluorescence‐based ·NO detection, confocal microscopy, and time‐resolved photoluminescence (TRPL) spectroscopy, we systematically investigate how P3HT‐NP internalization and photoactivation regulate intracellular ·NO dynamics and cellular metabolic pathways in Human Umbilical Vein Endothelial Cells (HUVECs). Interestingly, we elucidate a bidirectional (i.e., increase or decrease) and reversible modulation of ·NO levels, depending on illumination conditions. The underlying biological pathways are critically analyzed and discussed. Preliminary data also demonstrate that the tool is not specific to HUVECs, but can be extended to other endothelial models like murine microvascular cardiac endothelial lines.

Overall, our approach enables a light‐triggered and dynamic fine tuning of ·NO signaling in both directions, without genetic manipulation. These results may open new avenues for redox‐based therapeutic strategies, contributing to the emerging field of photo‐redox medicine.

## 2 Experimental Results

### 2.1 Synthesis and Characterization of Poly(3‐Hexyl‐Thiophene)‐Based NPs

The foundational hypothesis of this work is the opportunity to exploit the well‐known photocatalytic activity of P3HT NPs, as well as their ability to precisely modulate ROS levels in endothelial cells on demand, in order to control additional key redox signaling mediators. In particular, we target here ·NO, given its pivotal role in vascular physiology and therapeutics, and the current lack of tools enabling its reversible and spatiotemporally controlled modulation.

In this framework, employing P3HT‐based NPs with the highest possible photoelectrochemical efficiency offers a distinct advantage, as it may allow to broaden the dynamic range of ROS/ ·NO modulation, simultaneously reducing the required optical power density. Here, we employ a novel type of NPs, whose synthesis was recently reported [31], based on P3HT, as the photoelectrochemically active component, and a hole transporting material, namely poly(3,4‐ethylenedioxythiophene) polystyrene sulfonate (PEDOT:PSS), as a cyto‐compatible, electrically and ionically conducting polymer. These NPs, hereinafter referred to as 3P NPs, are synthesized via a double‐emulsion method, as summarized in the Materials and Methods (section 5.1) to the sake of completeness. As extensively documented in [31], the intermixing between P3HT and PEDOT:PSS leads to the formation of a ‘diffuse’ interface, with the formation of PEDOT:PSS islets within P3HT semiconductor. The introduction of the PEDOT:PSS/P3HT interface, which is active in the dissociation of charges photogenerated by the P3HT polymer, results in more efficient photoelectrochemical ROS generation at the NP/electrolyte interface, while NP size and optical absorption remain comparable in both homogeneous P3HT and heterogeneous P3HT/PEDOT:PSS systems. This result was obtained through photoelectrochemical experiments, optical absorption and dynamic light scattering, as part of a much broader characterization study in which 3P NPs were systematically, quantitatively compared with bare P3HT NPs [31].

To increase the relevance and range of applicability of the 3P NP architecture, we further enrich the structure by introducing a calcium phosphate (CaP) capping layer. This material has been shown to be suitable for subsequent functionalization with aptamers to enable targeting of specific cells, a key requirement for therapeutic applications [44]. Although in vivo studies are beyond the scope of the present work, engineering NPs with a CaP capping layer allows us to assess the reliability and impact of a system already compatible with future cell‐specific in vivo therapies.

**FIGURE 1:**
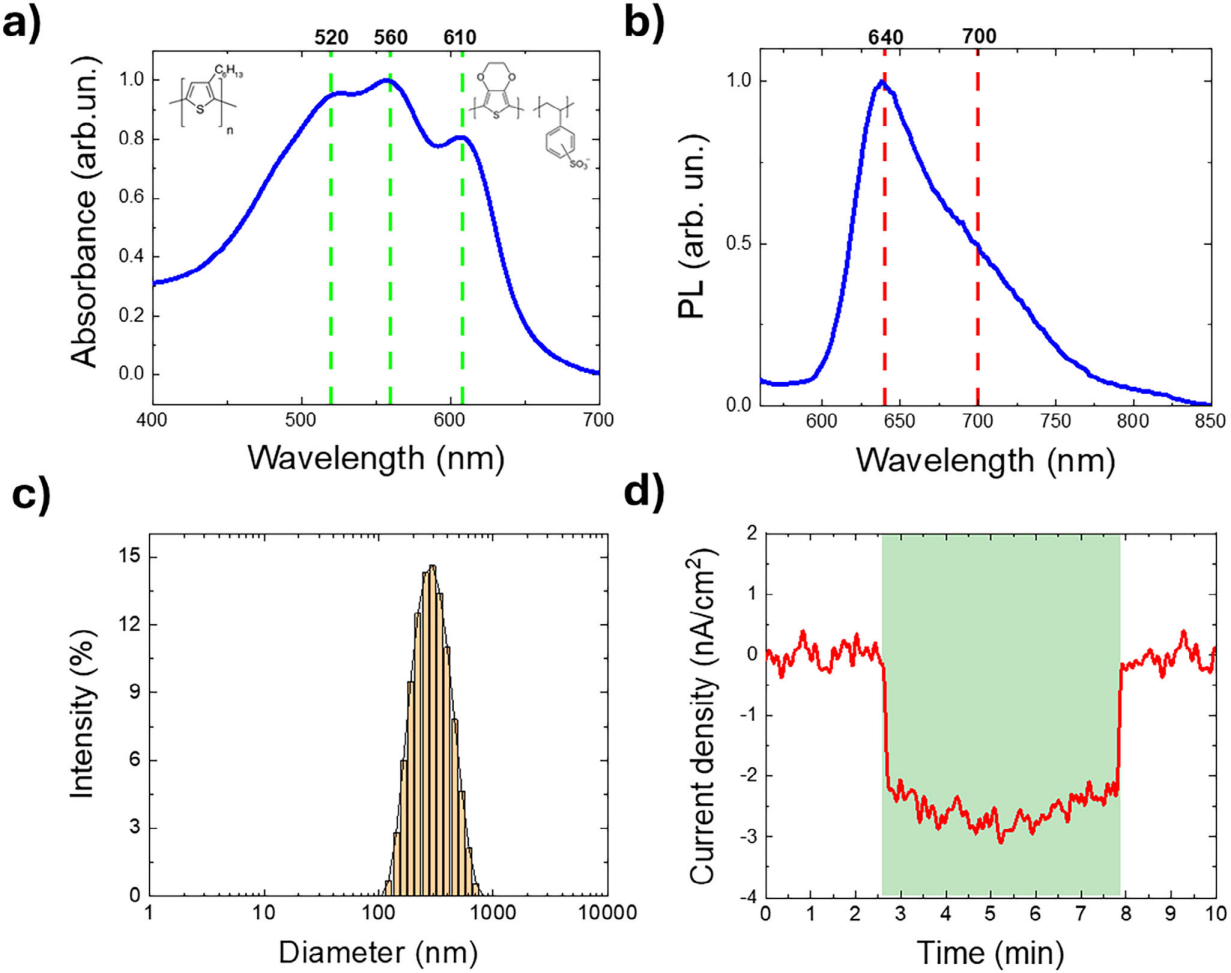
Characterization of 3P NPs aqueous colloidal dispersions. (a, b) Normalized UV–vis absorption and photoluminescence spectra. Laser excitation wavelength, 530 nm. (c) Representative size distribution obtained through DLS. The x‐axis is reported in logarithmic scale. (d) Photocurrent production of 3P NPs in aqueous dispersions. Photocurrent recorded in a 2‐electrode setup, at the Open Circuit Potential (OCP) in dark conditions (0.12 V vs reference electrode). Shaded green area represents the illumination (LED light source, emission peak at 530 nm, power density 38 mW/cm^2^).

Figure 1 displays main optoelectronic features of 3P NPs, of interest for the present work. The UV–vis and fluorescence emission spectra (Figure 1a,b, respectively) show features typical of P3HT NPs aqueous dispersions, with absorption peaks around 520, 560 and 610 nm and emission peak and shoulder at 640 and 700 nm, respectively [45, 46].

3P NPs hydrodynamic diameter was measured by Dynamic Light Scattering (DLS), obtaining an average value of 296 ± 66 nm and a mean Polydispersity Index (PDI) of 0.22 ± 0.04 (Figure 1c). This dimension falls in a size range, between approximately 100 nm and 330 nm, which was proven to be safe for the realization of photoactive interfaces between P3HT NPs and line cells, including human embryonic kidney [47] and immortalized human keratinocytes [48]. In all those cases, P3HT NPs were reported to efficiently internalize within the cell cytosol without crossing the nuclear barrier. In addition, the presence of the NPs did not impair cell proliferation, nor induced signs of cytotoxicity or long‐lasting cell membrane disruption, nor altered the physiological cell membrane passive electrical properties [47, 49]. 3P NPs of comparable dimensions were also reported to cross the cytosol membrane of HUVECs, localizing in a perinuclear region, without altering cell viability and proliferation capability [31].

3P NPs in aqueous colloidal dispersion maintain the capability to generate a photocathodic current, ascribed to oxygen reduction reactions occurring between photogenerated electrons and molecular oxygen dissolved within the medium, according to extensive literature data and accepted interpretation [31]. Importantly, detailed characterization of 3P NPs as compared to control, bare P3HT NPs showed that the introduction of the diffused interface between PEDOT:PSS multicores and P3HT leads to sizable improvement of the charge dissociation, thus considerably enhancing, by about +40%, the efficiency toward photoelectrochemical oxygen reduction and production of ROS within the cell cytosol, as compared to bare semiconducting NPs without PEDOT:PSS. This previous observation motivated the selection of 3P NPs in the present work. Photocathodic current was recorded with all the 3P NPs batches synthesized for this work, obtaining an average absolute value of about 2.5 nA/cm^2^ in the illumination conditions hereby considered (Figure 1d).

### 2.2 Bidirectional Modulation of Endothelial ·NO by NP Photostimulation

**FIGURE 2:**
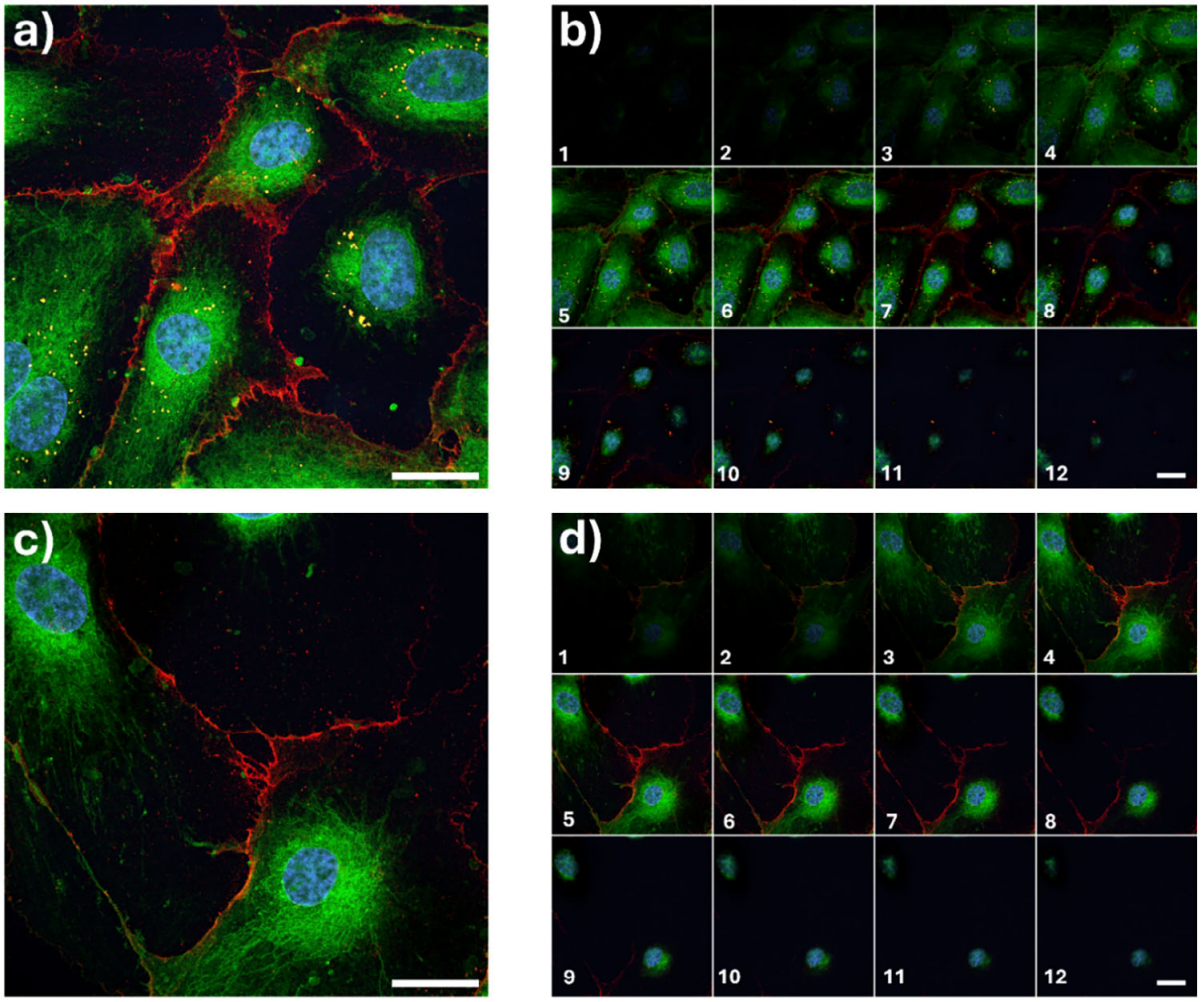
Immunofluorescence staining of in vitro HUVEC cultures, treated with 3P NPs (a,b) and NPs‐untreated controls (c,d). Nuclei, actin filaments and cytosol membranes are labelled with Hoechst 33342, phalloidin‐FITC and CD31‐FAB2 and are visible in blue, green, and red, respectively. 3P NPs can be visualized thanks to their intrinsic fluorescence (excitation/emission wavelengths: 561/650 nm) and have been colored in yellow. Images acquired at multiple focal planes demonstrate 3P NPs internalization within the HUVEC cytosol (panel b). Focal planes are captured from the top interface with the extracellular bath (z = 1) to the bottom of the cells (z = 12), with an interplane distance of 0.17 µm. Scale bar, 20 µm.

To assess the potential of 3P NPs to modulate ·NO in endothelial cells, we selected HUVECs as a physiologically relevant model of the human endothelium. HUVECs are widely recognized for their prominent eNOS activity and ability to produce ·NO, making them an appropriate system for investigating endothelial ·NO signaling pathways [50]. 3P NPs (O.D. = 0.1) were added to the cell culture 3 h after plating and rinsed after 6 h of incubation. Illumination with 530 nm LEDs was performed for 6 h with a pulsed protocol (100 ms ON / 900 ms OFF) at a power density of 6 mW/cm^2^. First, we evaluated the impact of 3P NPs, light stimulation and combined conditions on HUVECs proliferation, at three distinct time points, 1DIV, 3DIV and 7DIV after plating. Data do not evidence statistically significant differences with respect to control samples, namely NPs‐untreated cells maintained in dark condition (Figure S1). Effective uptake of 3P NPs within HUVEC cultures and their localization within the cell cytosol was assessed by means of confocal microscopy, by comparing NPs‐treated (Figure 2a,b) and untreated (Figure 2c,d) cells. Nuclei, cell membrane and actin filaments can be visualized by immunostaining with DAPI (blue), CD31 (red) and phalloidin (green), respectively; 3P NPs can be identified by taking advantage of their intrinsic fluorescence (yellow spots, Figure 2a,b). Z‐stack images, acquired at multiple focal planes spanning from the top to the bottom of the cell, demonstrate that 3P NPs cross the plasma membrane and localize within the cytoplasm, with no evidence of nuclear crossing. These findings are consistent with previously reported localization patterns of P3HT NPs in other cell models [30, 38, 47].

**FIGURE 3:**
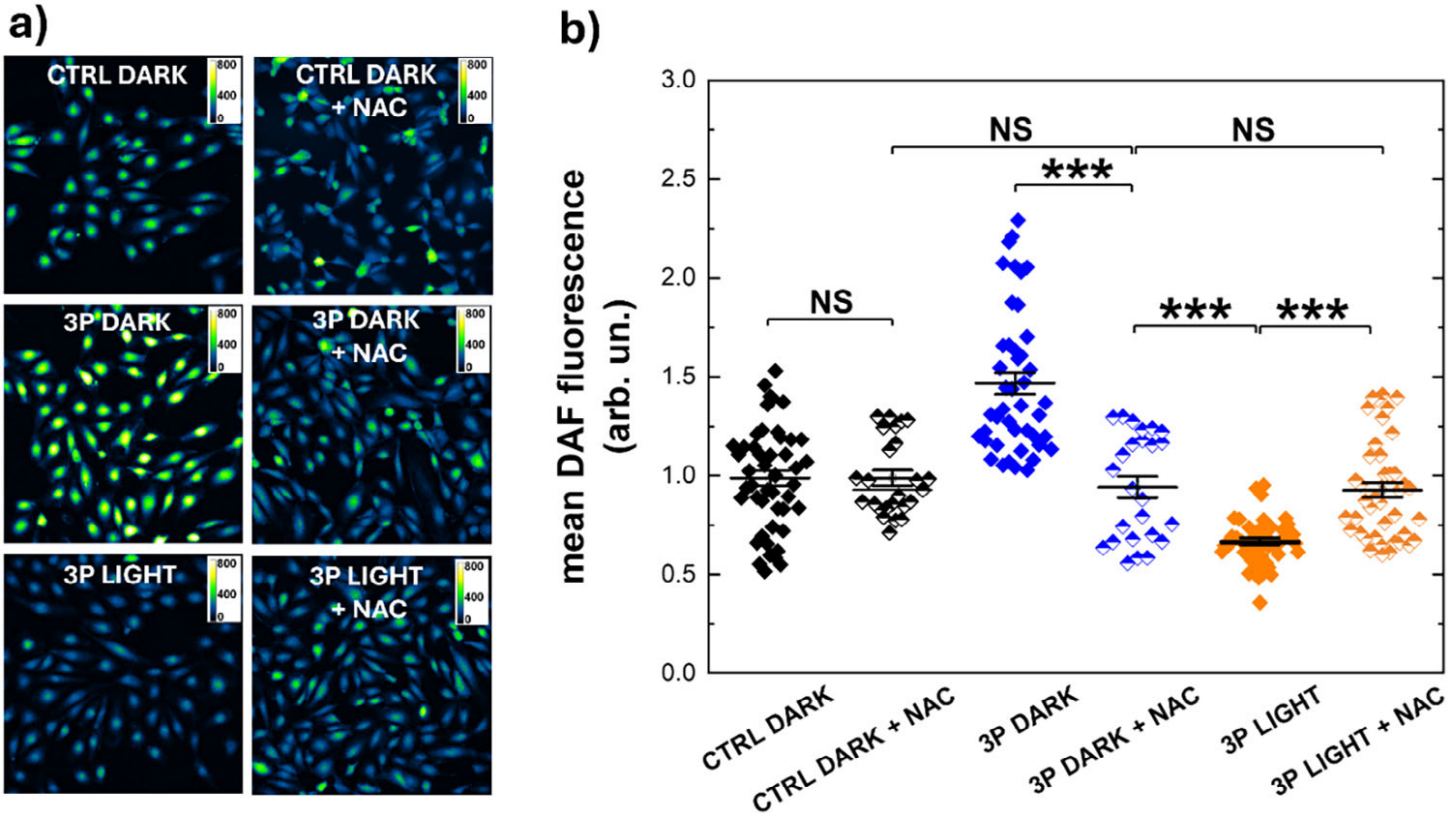
Intracellular ·NO concentration in HUVECs, evaluated by the cell permeant, ·NO‐sensitive fluorescent probe DAF‐FM‐DA. (a) Fluorescence images of representative fields for controls and NPs‐ treated samples (blue‐to‐white colormap). (b) Data representing the mean DAF fluorescence for each condition. 3P NPs and NAC treatment were added to cell culture 3 h after plating, while light stimulation (λ = 530 nm, 6 mW/cm^2^, 100 ms ON / 900 ms OFF, for 6 h) was performed the following day, prior to measurements. Each dot represents a field of view, with each field averaging at least 15 cells; total number of cells 500 < n < 700 for each condition. Data are represented as mean ± SEM, normalized to control dark. Statistical analysis was performed on three independent biological replicates using the Mann‐Whitney U‐test. Significance values: *** for *p* < 0.001.

Having assessed the localization of 3P NPs within the cytosol, we investigated their effect on intracellular ·NO levels, both in dark and following photoexcitation. To this goal, we employed the cell‐permeant fluorescent probe 4‐Amino‐5‐Methylamino‐2',7'‐Difluorofluorescein Diacetate (DAF‐FM‐DA) (see Materials and Methods section 5.5 for experimental protocol details). The probe, once delivered to the cell culture medium, passively diffuses across the plasma membrane and is hydrolyzed by esterases to yield DAF‐FM, which is then oxidized by ·NO to form a fluorescent triazole derivative. Thanks to its high emission quantum yield, DAF‐FM allows detection of ·NO even under resting conditions and enables semi‐quantitative monitoring of ·NO concentration changes in response to extracellular stimuli, allowing comparison between experimental conditions. Figure 3a shows representative fluorescence images of ·NO levels acquired in both control (NPs‐untreated, in dark condition) and NP‐treated samples, in dark or after NPs photoexcitation. The fluorescence signal, proportional to ·NO accumulation, is visualized using a blue‐to‐white colormap. We observe that the bare treatment with 3P NPs (not exposed to photoexcitation), turns into a significant ·NO increase, approximately +50% vs. control dark condition. On the contrary, 3P NPs photostimulation leads to a remarkable ·NO reduction, by about −55% vs. 3P dark condition and −30% vs. control dark condition. Notably, unlike previously reported photoactivated ·NO‐donor or photobiomodulation‐based approaches, the observed ·NO decrease under photostimulation represents an active reversal of intracellular ·NO levels below basal conditions, achieved within the same material system. Figure S2 reports the impact of the sole photoexcitation protocol on ·NO levels in NPs‐untreated HUVECs. In line with recent literature reports [13, 14, 51, 52], the control light condition shows a significant ·NO increase (∼ +80%) compared to control dark conditions. In brief, both NPs treatment and light exposure, independently applied, promote a remarkable ·NO increase. However, when combined they do not produce an additive effect; instead, they result in a ·NO decrease even below the basal levels observed in the control dark condition.

These results show that the ·NO response in NPs‐treated cells follows a bimodal pattern that is dependent on light activation, suggesting an interplay between NPs charge photogeneration and ·NO‐related signaling pathways.

Our previous work extensively demonstrated that photoexcitation of 3P NPs induces ROS generation, with an increase of hydrogen peroxide (H_2_O_2_) within the range of safe physiological concentrations [31]. Moreover, polymeric and inorganic NPs have shown to exert a bimodal pro‐/anti‐angiogenic behavior, with low ROS levels promoting and high ROS levels inhibiting angiogenic signaling, respectively [53, 54]. On the other side, the crosstalk between ROS and ·NO pathways is now largely documented in literature [55], especially in endothelial cell models, where it plays a crucial role in regulating angiogenesis and holds significant therapeutic potential.

Given this established interaction, and the observed light‐dependent bimodal modulation of ·NO in our system, we aimed to directly test whether ROS photogeneration is responsible for this effect. Thus, we repeated the experiment using N‐acetyl‐l‐cysteine (NAC), a highly efficient and widely employed ROS inhibitor [56]. In fact, NAC is a synthetic precursor of intracellular cysteine and glutathione (GSH), and it is known to inhibit ROS through various mechanisms [57]. Figure 3b shows that addition of NAC to NPs‐treated cell cultures deterministically leads to full restoration of ·NO to the basal concentration, in the case of both depleted (i.e., joint effect of NPs and photoexcitation, orange data points) and increased (i.e., bare effect of NPs in dark condition, blue data points) ·NO levels. As a control, any direct interaction between NAC and ·NO can be safely ruled out, since NAC delivery has no significant effect on control untreated samples, in dark (black data points).

Overall, the ability of NAC to consistently restore ·NO levels to basal values across all tested conditions underscores the critical role of ROS in modulating intracellular ·NO levels.

Together, these results unambiguously show that ROS play a key role in ·NO modulation. Experimental data do not allow for univocally identifying and quantitatively disentangling relative contribution of different ROS species; however, we tentatively propose an interpretative model in which the increase in intracellular ·NO observed in dark is likely mediated by ROS‐dependent activation of eNOS, rather than by a direct ·NO‐releasing effect of the NPs. Conversely, the decrease in intracellular ·NO observed upon photostimulation is attributable to elevated ROS levels, which likely promote rapid ·NO consumption by reactive oxidizing species and/or oxidative inhibition of NOS activity under high oxidative stress.

ROS appear here to be associated to the activation of inflammatory pathways following both bare NPs internalization, as documented in literature [58], as well as to P3HT photoelectrochemical activity toward oxygen reduction and subsequent modulation of the intracellular redox balance [30, 38].

We infer that ROS play a key role in NO modulation; however, the origin and modulation of ROS production may also be influenced by the physicochemical properties and internal morphology of 3P NPs themselves.

In fact, 3P NPs are characterized by a structured architecture, with a preferential localization of ionically and electronically conducting PEDOT:PSS islets within the NP bulk and of photoactive, semiconducting P3HT at the NP surface exposed to the electrolyte environment (see [31] for detailed analysis and characterization). The peculiar morphology of 3P NPs may potentially play a role in the interaction with HUVEC cell metabolism and modulation of redox signalling. Moreover, while PEDOT:PSS and P3HT, both based on thiophene repeated units, share a similar chemical composition, their different hydrophilicity, electronic transport mechanisms, and optoelectronic properties may also exert a non‐negligible impact on modulation of intracellular ·NO concentration. In order to disentangle and isolate a possible role of the PEDOT:PSS component (besides its obvious contribution in enhancing charge dissociation processes and subsequent increase of photo‐electrochemical efficiency), we carried out analogue experiments by using bare P3HT NPs.

As shown in Figure S3, internalization of P3HT NPs alone leads to an increase in ·NO levels, compared to untreated samples, while NPs photostimulation turns into ·NO relative decrease, compared to untreated control cells and treated cells in dark condition. These results qualitatively account for the key contribution of the P3HT photoactive component within 3P NPs in inducing light‐dependent, bimodal modulation of ·NO, and allow us to conclude that P3HT is the primary component responsible for the observed ·NO regulation, notwithstanding with the documented role of PEDOT:PSS in increasing the oxygen reduction reaction efficiency and ROS photogeneration capability.

A very preliminary, qualitative investigation was also carried out to assess the specificity of the observed ·NO regulation by 3P NPs. To this goal, we considered a second endothelial cell model, namely a murine microvascular cardiac endothelial cell line (H5V). We observed a behavior fully similar to the one reported for HUVECs: 3P NPs treatment nearly doubles ·NO levels compared to the control dark condition, while 3P NPs photostimulation induces a significant decrease relative both to the 3P dark condition and to the control dark condition (Figure S4).

In summary, the data reported so far, taken as a whole, show that:

1) Internalization of 3P NPs alone promotes a significant increase in endogenous intracellular ·NO levels;
2) Photostimulation of 3P NPs reduces intracellular ·NO concentration below basal levels;
3) Both the ·NO increase and decrease, when mediated by polymer NPs, are tightly linked with ROS generation, as demonstrated by their suppression following NAC administration. Thus, ROS appear as key signaling mediators in both directions of ·NO modulation;
4) The observed bimodal modulation of ·NO does not depend neither on the structural organization of 3P NPs, nor on electronic/ionic conductivity provided by the PEDOT:PSS component, nor on the specific endothelial model used. Instead, the results obtained thus far point to the photocatalytic activity of the P3HT component as the primary driver of the light‐dependent, bimodal modulation of ·NO levels.

### 2.3 3P NPs Induce Glycolytic Metabolic Shift of Endogenous Origin

In the context of intracellular modulation, especially when ROS and ·NO are involved, it is crucial to evaluate possible metabolic changes. Such analysis enables the assessment of whether ROS or ·NO‐induced signaling pathway modifications exert an influence on cellular energy metabolism. Being able to quantify these metabolic shifts, in particular, is essential to determine whether intracellular signaling modulation remains within the bounds of physiological *eu*stress or progresses toward a state of oxidative or nitrosative *di*stress [40].

Here, we evaluate the metabolic state of nicotinamide adenine dinucleotide (NADH), as a crucial cofactor in cellular metabolism participating in various energy‐producing pathways and oxidative stress dynamics [59]. NADH autofluorescence lifetime detection can be used in live cells as label‐free, non‐destructive tool to investigate the cellular metabolic state. Variations in NADH lifetime correlate with metabolic profiles, providing deeper insights into how cells adapt to changes and stressors [60]. To quantitatively assess NADH fluorescence decay rates, we employ TRPL spectroscopy. This technique provides single‐cell resolution of the cellular metabolic state and offers valuable insights into the complex dynamics of metabolic processes, without requiring the use of chemical probes or drugs [61].

In cells, NADH exists in two primary forms: free (unbound) and enzyme‐bound, each one characterized by distinct fluorescence lifetimes. The free form exhibits a fast decay (∼200 ps), while the bound form has a significantly longer decay (∼2 ns) [62]. By analysing NADH fluorescence in the perinuclear region of cells, we can estimate the relative abundance of both free and enzyme‐bound NADH, providing insights into the metabolic state of the cell. Specifically, an increase in the proportion of free NADH reflects a shift toward glycolytic metabolism, whereas a decrease indicates enhanced oxidative phosphorylation (OXPHOS) activity [63]. As NADH fluorescence arises from a mixture of both forms, their relative contributions can be reliably correlated with the cell's metabolic state.

**FIGURE 4:**
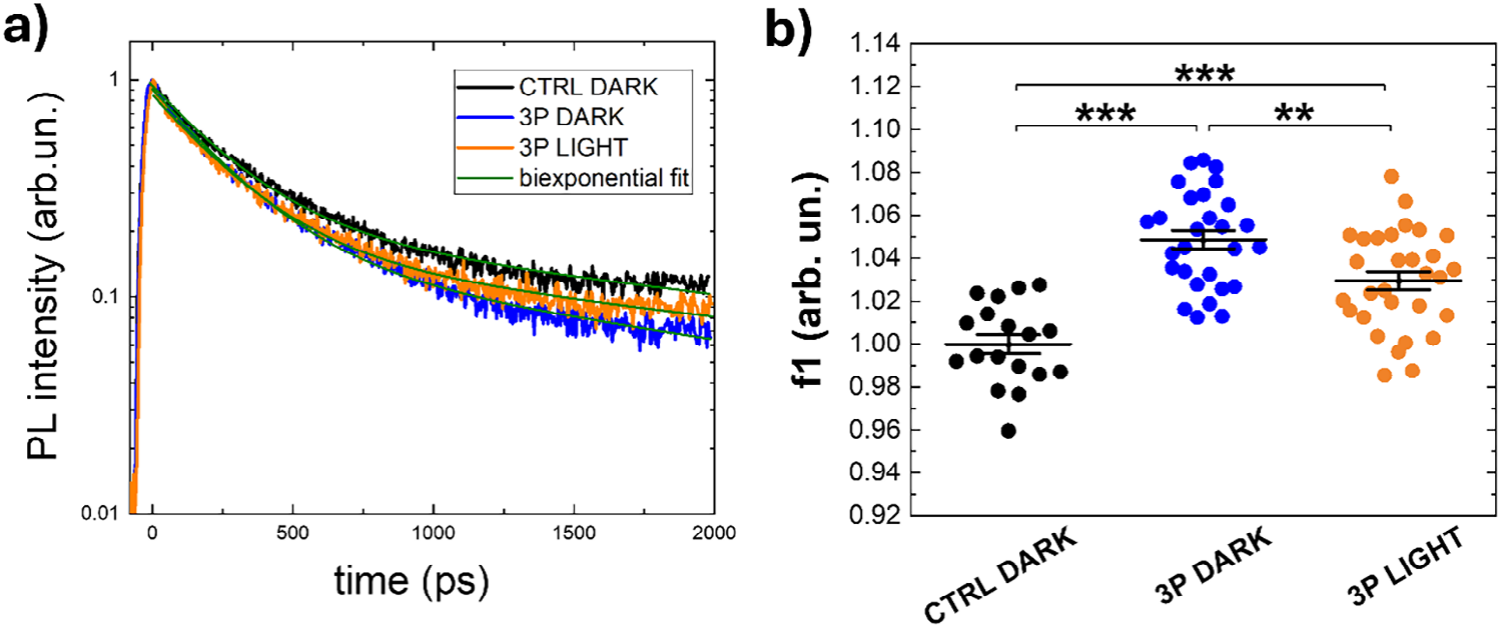
TRPL analysis of NADH metabolism in HUVECs. (a) Representative NADH fluorescence decay curves of the three experimental conditions. 3P NPs were added to cell culture 3 h after plating, while light stimulation (λ = 530 nm, 6 mW/cm^2^, 100 ms ON / 900 ms OFF, for 6 h) was applied the following day, prior to measurements. Decay traces were fitted with a biexponential model to extract the lifetimes of free and enzyme‐bound NADH. (b) Quantification of the fractional contribution of free NADH (*f*_1_) derived from the biexponential fit across different conditions. Each data point corresponds to an individual cell. Values are represented as mean ± SEM, normalized to control dark. Statistical significance determined by Mann‐Whitney U‐test over three independent biological replicates. Significance levels: *** for *p* < 0.001; ** for *p* <0.01.

Figure 4a shows representative NADH photoluminescence dynamics recorded in control, NPs‐untreated cells (black curve), as well as NPs‐treated samples, in dark (blue curve) or exposed to the photostimulation protocol (orange curve). To distinguish between free and bound NADH, fluorescence decay curves were fitted using a biexponential model (see Materials and Methods, section 5.6 for details) (green curves in Figure 4a). The resulting parameter, *f*_1_, represents the fractional contribution of free NADH, and its values for different cells are reported in Figure 4b.

Interestingly, treatment with 3P NPs, whether photoexcited or not, overall increases the free NADH pool as compared to untreated controls, in a statistically significant manner. We infer that NPs internalization leads to a slight metabolic shift (< 10%) toward glycolysis; the latter, however, remains within the range typically associated with redox‐mediated *eu*stress, without exceeding thresholds indicative of pathological metabolic alteration [64, 65]. Upon photostimulation, we observe a reduction in *f*_1_ value compared to non‐illuminated NPs, indicating a relative shift back toward OXPHOS and confirming the bidirectional effect enabled by polymer NPs in dark or upon photostimulation. However, the magnitude of the light‐induced metabolic shift, while pointing to the opposite direction, is weaker than the effect of NPs internalization in dark, resulting in a net shift toward a glycolytic behavior (∼3%) compared to untreated dark conditions. This incomplete restoration toward OXPHOS may represent a protective feedback response to the elevated ROS levels generated under light [66]. As shown in Figure S5, light exposure in absence of NPs does not produce any significant change in HUVECs metabolic state, indicating that the ·NO increase observed in the control light condition is not causally linked, per se, to a metabolic shift.

The glycolytic metabolic shift observed upon NPs administration may, in principle, be driven either by endogenous or exogenous ·NO sources, both of which can be interconnected with a ROS increase. The localization of NPs within the cell cytosol suggests that endogenous ·NO production is the most probable pathway; however, a contribution from a secondary, exogenous mechanism cannot be completely excluded at this stage. TRPL technique offers the opportunity to unequivocally and efficiently solve this doubt, as it provides direct information on the cell metabolic status while eliminating the need for chemical probes and allowing drugs to be used as pure stimuli. In other words, chemical stimulation and detection can be completely disentangled with this approach. Leveraging this advantage, we carried out TRPL measurements on HUVECs treated either with exogenous H_2_O_2_, at concentrations well within the *eu*stress range, or with S‐Nitroso‐N‐Acetylpenicillamine (SNAP), a well characterized ·NO donor that releases ·NO in aqueous solution [67]. As shown in Figures S6 and S7, both H_2_O_2_ and SNAP treatment induced a metabolic shift toward OXPHOS relative to control conditions. This response contrasts with the glycolytic shift observed following NP internalization, thus highlighting a fundamental difference between exogenously supplemented ROS/·NO and their endogenously generated counterparts, in terms of their impact on cellular metabolism. These findings further support the hypothesis that the ROS/·NO increase following NP internalization arises from intracellular regulatory mechanisms, rather than passive delivery from external sources.

### 2.4 Identification of ·NO Sources via eNOS and iNOS Immunostaining

To refine our understanding of the mechanisms leading to ·NO modulation by 3P NPs, we investigated the expression of the main nitric oxide synthase isoforms by carrying out immunofluorescence studies. In endothelial cells, ·NO is synthesized predominantly by two NOS isoforms: eNOS, typically activated by physiological stimuli and calcium influx, and iNOS, which is transcriptionally upregulated under oxidative stress [4, 67].

**FIGURE 5:**
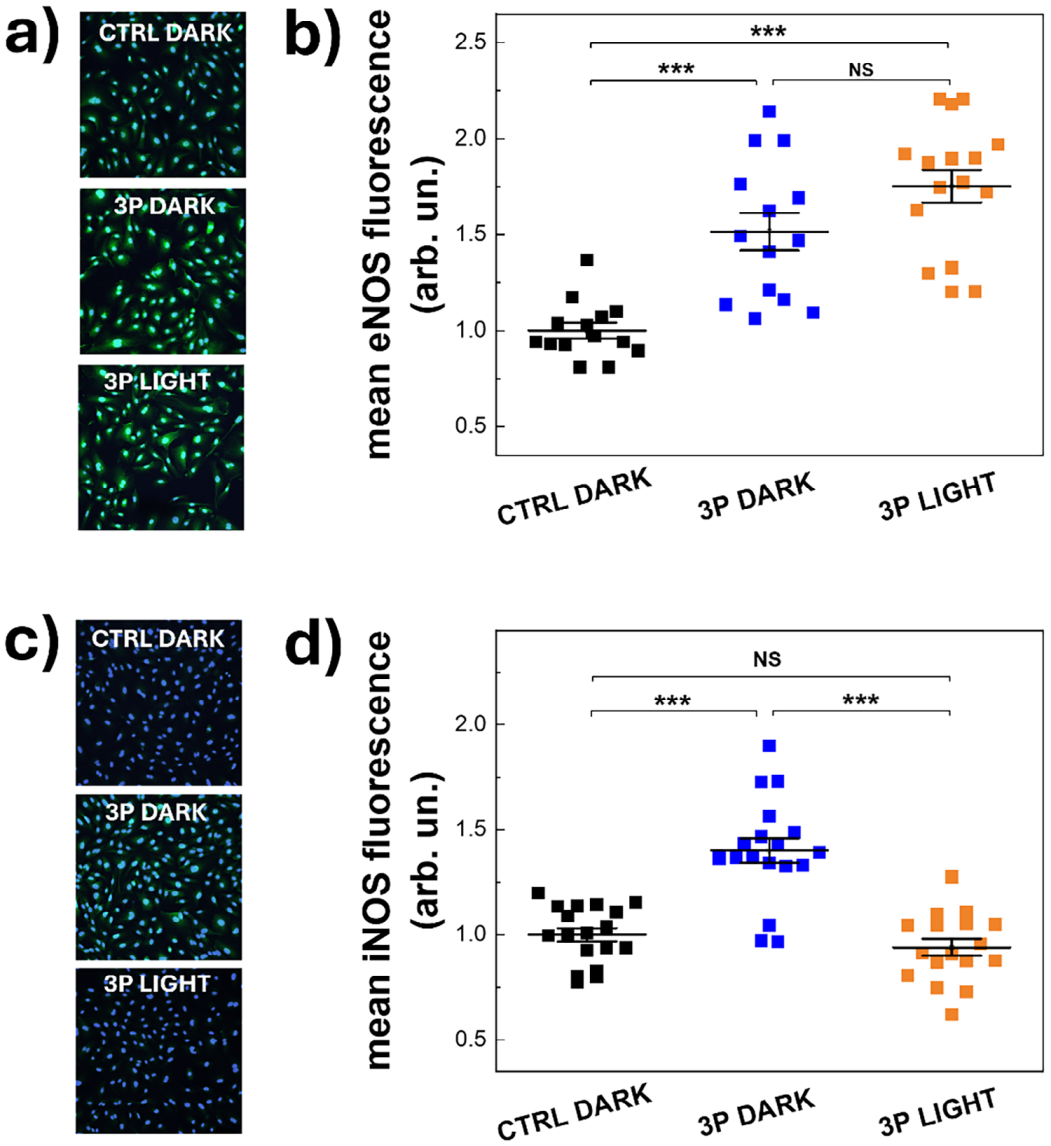
Immunofluorescence analysis of eNOS and iNOS expression in HUVECs. Fluorescence images of representative fields for control and 3P‐treated cells, stained with (a) eNOS or (c) iNOS (green) antibodies; nuclei are counterstained with Hoechst (blue). Quantification of (b) eNOS and (d) iNOS expression under different treatment conditions. Each data point represents a field of view, each field averaging at least 15 cells. Light stimulation (λ = 530 nm, 6 mW/cm^2^, 100 ms ON / 900 ms OFF, for 6 h) was performed the day after plating, prior to measurements. Data are represented as mean ± SEM values, normalized to control dark. Statistical analysis: one‐way ANOVA with Tukey's post hoc test. Significance levels: ***p < 0.001.

So, we performed immunostaining to assess both eNOS and iNOS expression under different treatment conditions, as reported in Figure 5. We observed a significant increase in eNOS expression compared to the basal value (∼+50%) in both NP‐treated conditions, with or without light photostimulation (Figure 5a). This upregulation of eNOS may represent a compensatory response to moderate redox perturbations induced by NPs internalization. Sub‐cytotoxic ROS levels are known to activate redox‐sensitive transcription factors, that is, Nrf2, and calcium‐dependent pathways, that is, Krüppel‐like factor, both of which can promote eNOS transcription aiming at preserving endothelial function under *eu*stress [7, 68]. As shown in Figure 5b, cells treated with 3P NPs in the dark also exhibited a significant increase (∼+40%) in iNOS expression compared to untreated controls, which may be driven by the redox‐sensitive transcription factor NF‐κB [69]. This upregulation aligns well with the elevated ·NO levels and the metabolic shifts toward glycolysis, consistent with the activation of a ROS‐driven inflammatory pathway in response to NP internalization. Upon NPs photostimulation, iNOS expression was markedly reduced, returning to values comparable to untreated controls. This light‐induced suppression mirrors the observed decrease in ·NO levels and the metabolic reversion toward OXPHOS, and is compatible with a feedback mechanism caused by higher levels of exogenous ROS generated by NP photostimulation, possibly by NF‐κB modulation and subsequent iNOS transcription [70].

Taken together, these results suggest that NP internalization, in the absence of photostimulation, induces an upregulation of both eNOS and iNOS expression, potentially enhancing ·NO production via a ROS‐dependent signaling pathway. Photostimulation triggers a protective response in which eNOS expression is maintained while iNOS is downregulated, possibly through ROS‐mediated inhibition of NF‐κB. We propose that this light‐dependent switch in NOS isoform expression may represent a key mechanism contributing to the bidirectional control of endothelial ·NO levels.

Building on these findings, it is also important to consider the effects of light treatment alone, without NPs internalization, on NOS isoform expression in endothelial cells. As indicated in Figure S8, light treatment alone leads to a moderate increase in eNOS expression, while leaving unchanged iNOS levels, as compared to dark controls. The selective upregulation of eNOS without iNOS induction supports the interpretation that light alone elicits a non‐inflammatory, physiological ·NO response, distinct from the redox mechanism triggered by NPs internalization.

## 3 Discussion and Conclusion

·NO is a multifaceted signaling molecule whose biological effects are highly concentration‐dependent, acting either as a protective regulator or as a contributor to oxidative stress and cardiovascular disorders [1]. Emerging evidence has highlighted that the biological outcome of ·NO signaling is critically governed by its steady‐state intracellular concentration, which determines whether ·NO acts in a protective, regulatory role or contributes to pathological processes.

Currently, several methods enable a unidirectional modulation of intracellular ·NO concentration: a number of ·NO‐releasing platforms and donors, able to precisely control the amount of ·NO released at target sites have been designed. These include small ·NO donor molecules, polymeric nanoparticles, dendrimers, liposomes [42, 71, 72]. Unfortunately, the majority of these systems are not able to precisely regulate the duration and/or the magnitude of ·NO release in situ, which could lead to aberrant ·NO signaling in the target tissue and exacerbating tissue damage [71]. Several stimuli‐responsive nanomaterials have been developed to release ·NO only at therapeutically relevant sites and times [73]. In particular, the use of photoactivated NPs has attracted significant interest due to their superior spatial and temporal resolution compared to pharmacological ·NO donors [74]. Reported systems include Platelet Membrane‐Coated PLGA NPs, activated by ultrasounds, SMA‐BmobaSNO, sensitive to NIR light, gold NPs, sensitive to pH or light [41], S‐nitroso‐N‐acetylpenicillamine (SNAP) and S‐nitrosoglutathione (GSNO) ·NO donors encapsulated within polydimethylsiloxane (PDMS) films (PDMS‐SNAP and PDMS‐GSNO, respectively), activated by UV–vis light [75], and SNP@MOF@Au‐Mal nanogenerators, sensitive to NIR light [76]. Another strategy is based on electrochemically‐activated ·NO generating/release systems, like iron‐sulfide nanoclusters, catalyzing NO generation from benign sodium nitrite in the presence of modest electric fields [77].

Nevertheless, although these novel ·NO‐donor platforms activated by physical stimulation can be engineered to selectively release ·NO at target sites in a temporally precise manner, they still lack bidirectionality. This is critical not only for the on‐demand initiation and termination of ·NO release, but also to reduce its concentration in a non‐invasive manner.

Bidirectional modulation of endothelial‐dependent ·NO release may offer a versatile, advanced strategy for targeting vascular function and related pathologies that require a dynamic fine‐tuning of ·NO levels. This approach may involve an initial phase of ·NO enhancement, followed by controlled inhibition to achieve the desired therapeutic outcome. To fully harness the benefits of both nanotechnology and light, we propose a strategy that disentangles the effects of the nanomaterial and light stimulation. Specifically, we use photoactive polymer NPs to stimulate endogenous ·NO production, followed by the use of light as regulatory tool to achieve a reversible, bidirectional modulation of ·NO levels.

To this goal, we select composite 3P NPs based on P3HT and PEDOT:PSS as semiconducting and conducting materials, respectively, which show superior photoelectrochemical performance and excellent cytocompatibility. We primarily focused our attention on the most widely employed model for investigating human endothelial biology [78]. Although HUVECs do not encompass the full heterogeneity of endothelial cell types present in vivo, namely the expression of the specialized endothelial barrier characteristics typical of brain microvascular endothelial cells, they provide a robust and well‐established system for studying vascular endothelial properties and the principal biological pathways involved in endothelial function [79]. To confirm that the effects exerted by 3P NPs could be extended to other endothelial cell types, we then assessed whether NP internalization affects ·NO levels in the murine microvascular cardiac endothelial cell line H5V, which has been widely used to investigate the role of endothelial cells in cardiac disorders [80]. We demonstrate that NPs internalization leads to a robust enhancement of intracellular ·NO levels in both HUVECs and H5V cells. In contrast, upon photostimulation, NPs induce a reduction in ·NO concentration below basal levels, enabling a spatially selective, bimodal ·NO modulation. By using NAC as a well‐established ROS scavenger, we provide strong evidence for a causal link between ·NO modulation and ROS generation under both dark and light conditions.

Taken together, our results support a dual, ROS‐mediated regulation of intracellular ·NO, in which the biological outcome depends on the magnitude of the generated ROS. Under dark conditions, NP internalization induces mild oxidative stress, which is known to activate redox‐sensitive signaling pathways and promote NOS activity, resulting in increased endogenous ·NO production. In contrast, upon illumination, photoexcited NPs generate substantially higher ROS fluxes. Under these conditions, intracellular ·NO levels decrease. It should be noticed that, while the DAF‐FM probe used in this study is widely accepted for qualitative, relative measurements of intracellular ·NO detection due to its relatively high specificity, it cannot provide absolute ·NO quantification. This is beyond the scope of the present paper, and should be interpreted as a relative indicator of intracellular ·NO‐dependent nitrosative activity. Nevertheless, DAF‐FM measurements were systematically complemented with all appropriate controls (light only, NP only, and use of SNAP as an exogenous ·NO donor) and with additional, independent measurements (NOS isoform immunostaining, ROS scavenging experiments, and label‐free metabolic analysis), which altogether provide converging and mechanistically consistent evidence of bidirectional ·NO modulation.

In both dark and light conditions, a causal role of ROS is unambiguously evidenced. Although individual ROS species were not directly resolved in this study, the data are consistent with a qualitative shift in dominant ROS species between the two regimes. Dark conditions likely involve endogenous species, such as H_2_O_2_, acting as signaling mediators, whereas photostimulation induces the additional formation of exogenous, short‐lived, highly reactive species (e.g., superoxide‐derived oxidants), which are capable of consuming ·NO.

Then, to gain deeper insight into the biological and physicochemical mechanisms driving bimodal ·NO modulation and involving ROS as key players in both dark and light conditions, we focused our analysis on the study of the intracellular metabolism. TRPL was employed here as a label‐free, non‐destructive technique to measure NADH fluorescence lifetimes, providing a semiquantitative estimation of metabolic shifts between glycolysis and OXPHOS. We show that, in the absence of light, NP internalization induces a metabolic shift toward glycolysis, while upon photostimulation there is a partial reversal of this effect, and cells exhibit increased OXPHOS activity. However, it is important to note that even under illumination, the metabolic state remains moderately shifted toward glycolysis when compared to control dark conditions. These results are in agreement with previous observation that bare P3HT NPs internalization determines an increase in the GSH regeneration [38]. This shift in the redox balance may be in turn associated with the enhancement of glycolytic activity observed in this work. Recent literature, reporting that elevated glycolytic activity promotes more efficient GSH regeneration, while GSH status can, in turn, feedback to modulate glycolysis [81], is in good agreement with this interpretation.

**FIGURE 6:**
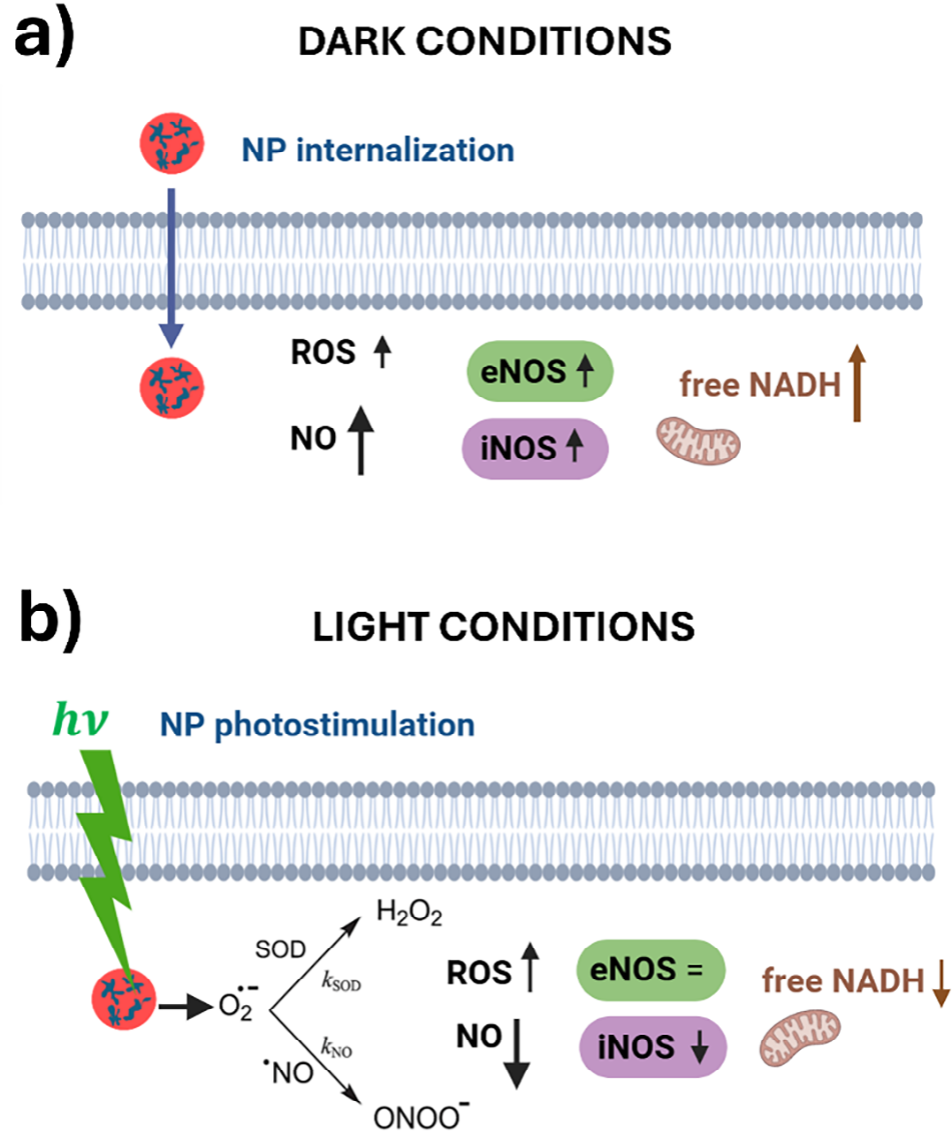
Schematic representation of the signaling cascade activated by 3P NP internalization and subsequent photoexcitation. (a) Under dark conditions, internalization of 3P NPs leads to an increase in intracellular ROS, which in turn induces a significant upregulation of iNOS and eNOS expression. This dual activation results in elevated ·NO levels, correlating with a shift toward glycolysis, as evidenced by increased free NADH in the cytoplasm. (b) Upon photostimulation, photoexcited NPs generate $O_{2}^{-}$, which scavenges ·NO, forming peroxynitrite, and further converts into ***H***_2_ ***O***_2_. The further increase in ROS levels by NPs photoexcitation contributes to the suppression of iNOS expression, while eNOS expression remains elevated. This mechanism is also accompanied by a partial reversal of the metabolic phenotype toward oxidative phosphorylation, reflected by decreased free NADH.

Taken together, these findings support the existence of two distinct regulatory pathways modulating intracellular metabolism, under dark and illumination conditions (Figure 6). We thus investigate the cellular sources of ·NO, since they are expected to provide useful information about the underlying modulation pathways. To this goal, NOS isoforms immunostaining was carried out. Interestingly, the glycolytic shift in metabolism observed following NP internalization under dark conditions is accompanied by a significant upregulation of both eNOS and iNOS.

Figure 6a schematically summarizes the main experimental observations and hypothesized signaling cascades upon NPs internalization and in absence of photoexcitation. The increase in iNOS expression is tentatively attributed to a moderate intracellular accumulation of ROS following NP uptake. ROS are known to activate redox‐sensitive transcription factors, such as NF‐κB, which may in turn promote iNOS transcription as part of an Inflammatory‐like stress response [69]. The concurrent upregulation of eNOS likely represents a compensatory, pro‐homeostatic response to *eu*stress redox imbalance. Coherently with this hypothesis, sub‐cytotoxic ROS levels and mild shifts in intracellular calcium signaling have previously been shown to stimulate eNOS expression [7] to regulate endothelial function.

This supports a picture where ROS generated upon NP internalization trigger redox signaling cascades, leading to increased iNOS and eNOS and, as a major outcome, sustained ·NO production. Thus, the observed increase in intracellular ·NO levels following NP internalization appears to be at least partially attributable to the activation of inflammatory signaling pathways [58]. The associated, minimal upregulation of glycolysis does not impair cell proliferation, suggesting that the metabolic and inflammatory responses remain well within physiological *eu*stress thresholds. In brief, while iNOS‐mediated ·NO production is indicative of an activated state, it never reaches levels associated with cytotoxicity or metabolic *di*stress.

Furthermore, our findings suggest that the ·NO increase induced by NP internalization is of endogenous origin. This conclusion is supported by comparative TRPL measurements performed on cells treated with SNAP, a well‐established ·NO donor whose effect on intracellular ·NO increase, as measured by DAF fluorescence, is well documented [67, 82]. Notably, SNAP treatment induced a metabolic shift toward OXPHOS, in contrast to the glycolytic shift observed following NP internalization. This metabolic shift highlights the distinction between exogenous ·NO supplementation and NP‐induced, endogenously generated ·NO, further supporting the distinct intracellular nature of NP‐induced intracellular ·NO increases compared to exogenous ·NO supplementation.

Supporting this interpretation, the iNOS and eNOS data provide additional support for the involvement of endogenous ·NO synthesis pathways. These findings reinforce the physiological relevance of our results, suggesting that modulating ·NO levels through NP‐mediated intracellular mechanisms may represent a more controlled and homeostatically compatible strategy, maintaining ·NO concentrations within *eu*stress thresholds more effectively than pharmacological, exogenous approaches.

Figure 6b reports a sketch of the hypothesized signaling mechanisms activated by 3P NPs photoexcitation. Photoelectrochemical activation of NPs under illumination modulates ·NO signaling through different intracellular pathways compared to control dark conditions. Our data show that photostimulation of 3P NPs leads to a decrease in ·NO concentrations in both endothelial cell models, with levels returning to baseline in NAC‐pretreated cells. Considering previous reports on ROS formation upon P3HT NP stimulation [29, 47], these findings support a mechanism in which superoxide ($O_{2}^{-}$) photoelectrochemically generated in the intracellular aqueous environment reacts with ·NO to form peroxynitrite (ONOO^−^) [83]. Thus, we can infer that the observed ·NO decrease as measured by DAF is driven by its rapid consumption through a reaction with photostimulation‐induced $O_{2}^{-}$:

$$\mathrm{NO}+O_{2}^{-}\to \mathrm{ONO}O^{-}$$

The reaction between $O_{2}^{-}$ and ·NO forming ONOO^−^ is both kinetically and thermodynamically more favourable than the reaction of $O_{2}^{-}$ with superoxide dismutase (SOD), thereby favoring peroxynitrite formation over H_2_O_2_ production [84]. However, accurately quantifying the exact amount of ONOO^−^ formation remains challenging, depending on factors such as lifetime, local concentration, and distance of the precursors which dictate the rate of peroxynitrite formation [84]. Undoubtedly, a portion of the $O_{2}^{-}$ generated upon NP photostimulation is likely still converted into H_2_O_2_ [38, 47] and this further contributes to the metabolic shifts observed by TRPL under light conditions.

To further investigate the role of H_2_O_2_ in mediating metabolic shifts, we performed TRPL measurements on cells pretreated with 1 µM H_2_O_2_, a concentration within the *eu*stress range and comparable to the levels of H_2_O_2_ expected to be generated by SOD after NP photostimulation [40]. The results revealed a pronounced metabolic shift toward OXPHOS, mirroring the response observed in cells treated with 3P NPs under light stimulation. These results are consistent with the hypothesis that the metabolic shift induced by NP photostimulation is, at least partially, mediated by exogenously produced ROS, such as H_2_O_2_.

The consistency of this response with those observed under light‐activated NP conditions, but not in the dark, reinforces the role of ROS generated upon NP photoactivation as key drivers of the observed metabolic modulation.

Moreover, these findings align with previous reports describing a bimodal modulation of ·NO levels in response to varying ROS concentrations. Specifically, low concentrations of H_2_O_2_ have been shown to enhance ·NO production, while higher concentrations lead to its suppression [85]. This bimodal response provides a mechanistic framework that may help explain the light‐dependent ·NO regulation observed in our system, linking ROS dynamics to both redox signaling and metabolic shifts.

Regarding eNOS expression upon NPs photostimulation, no significant change was detected with respect to NPs dark values. This suggests that eNOS expression is not the primary driver of the dynamic changes in intracellular ·NO decrease upon NPs photostimulation. Instead, our results point to iNOS and ROS as the dominant regulators of ·NO decrease. In addition to modulating NOS isoforms, exogenous ROS produced under NPs photostimulation influence cellular metabolism, triggering a partial shift toward OXPHOS, as seen in TRPL data. This shift is likely part of the same protective redox feedback mechanism: by adjusting both ·NO synthesis via iNOS downregulation and energy metabolism, the cell minimizes oxidative and nitrosative stress, while preserving metabolic flexibility.

In conclusion, this work highlights the potential of 3P NPs as an innovative platform for reversible, light‐controllable, bimodal modulation of ·NO in endothelial cells. The system functions as a dual‐phase therapeutic tool, enhancing ·NO production under dark conditions and downregulating it upon photostimulation.

The ability of our approach to regulate endothelial responses in a temporally precise and reversible manner opens avenues for its application in various redox‐related pathologies. Importantly, it ensures that ·NO is beneficially harnessed during early protective phases, while avoiding prolonged or excessive exposure that could contribute to inflammation or oxidative stress in later stages.

As a proof of concept, we have already demonstrated the effectiveness of 3P NPs in achieving bimodal regulation of angiogenesis through light stimulation [31], showcasing the translational potential of this approach in vascular regenerative medicine.

By integrating material‐driven intracellular stimulation with light‐triggered regulation, our approach introduces a powerful, dynamic modality for controlling ·NO signaling with high spatiotemporal resolution. This platform may prove particularly valuable in pathologies where precise, reversible ·NO modulation is essential, such as vascular dysfunction, tumor angiogenesis, vascular inflammation, wound healing, and endothelial barrier regulation, among others. Given the intricate role of ·NO in physiological signaling, the system presented here holds value not only as a therapeutic tool, but also as a research platform to better elucidate the complex signaling networks underlying vascular health and disease. The evidence that the green light‐dependent bidirectional modulation of ·NO release by P3HT‐based NPs can be achieved not only in HUVECs, but also in H5V cells, suggests that this approach will prove effective at increasing endothelial ·NO levels throughout the vascular bed. Moreover, light‐responsive semiconducting NPs are expected to enable the on‐demand control of ·NO release/scavenging in the other cellular components of the cardiovascular system that express both eNOS and iNOS, for example, cardiomyocytes, vascular smooth muscle cells and pericytes.

Overall, we believe that the results of this study provide a reliable tool for fundamental in vitro studies, and at the same time a solid foundation for developing in vivo, redox‐based strategies.

The path toward therapeutic applications of polymer NP‐mediated bimodal ·NO modulation should include, on one side, an improved rational design of nanomaterials, for example, by employing red and NIR light sensitive conducting polymers, properly functionalized to selectively target cells/tissues. We expect that similar outcomes may be obtained by considering other in vitro cell models, where ·NO modulation is known to play a key regulatory role. On the other hand, diverse preclinical applications may be investigated. The strategy we described here has been designed to rescue ·NO signalling in dysfunctional endothelium, which is the hallmark of cardiovascular disorders, including coronary artery disease, ischemic heart disease, brain stroke, systemic hypertension, diabetes, and pulmonary artery hypertension.

## 4 Materials and Methods

### 4.1 3P NPs and P3HT NPs Synthesis

#### 4.1.1 3P NPS

3P composite NPs were synthesized using a two‐step mini‐emulsion approach, followed by the addition of a calcium phosphate layer. Initially, 0.2 mL of a 1.1% PEDOT:PSS (Sigma‐Aldrich) aqueous solution was mixed with 0.2 mL of a 0.1% cetyltrimethylammonium bromide (CTAB, Sigma Aldrich) dispersion in water and stirred for 10 min at 30°C. Subsequently, 2 mL of P3HT dissolved in chloroform (5 mg/mL, Sigma‐Aldrich, Mw 20–45 kDa) was added to the aqueous phase under vigorous stirring, achieving a 5:1 organic‐to‐aqueous volume ratio, and forming the first water‐in‐oil (w/o) emulsion. The latter was then stirred at room temperature for 4 h and treated with a tip sonicator (tip width:1/8;″ 70% amplitude; cycle parameters: 3 min ON, 1 min OFF, 2 min ON). Following this, 10 mL of a 0.05% sodium dodecyl sulfate (SDS) aqueous solution, acting as a stabilizing agent, was added to the emulsion and stirred at 30°C for 1 h. A second round of tip ultrasonication was performed under the same conditions, leading to the formation of a stable water‐in‐oil‐in‐water (w/o/w) double mini‐emulsion. The chloroform solvent was then allowed to evaporate by stirring the mixture at 40°C for 3h, resulting in the formation of the composite nanoparticles. The latter were filtered through a 0.2 µm PES membrane and diluted 1:20 in ultrapure water. The filtered nanoparticle dispersion was then treated with the following reagents: 0.90 mL of CaCl_2_ (33.3 mM), 1.00 mL of Na_3_C_6_H_5_O_7_ (133.3 mM), 0.25 mL of Na_2_HP0_4_ 1(66.7 mM), and 0.85 mL of ultrapure MilliQ water for volume adjustment. A vortex mixer was employed to mix the dispersion after each addition. The mixture was then placed in a temperature‐controlled water bath at 60°C for 1h, under constant magnetic stirring (∼300 rpm). This step allowed for the complete functionalization of the P3HT‐based composite nanoparticles with a calcium phosphate outer layer.

#### 4.1.2 P3HT NPs

P3HT NPs were synthesized by flash nanoprecipitation of 1 mL P3HT, solubilized at a concentration of 1 mg/mL in tetrahydrofuran (THF) at 60°C for 1 h, into 10 mL of Milli‐Q water under stirring at 1300 rpm and 85°C. After 10 s, the nanoparticles dispersion was transferred to a beaker to evaporate the THF at 50°C under stirring for 1.5 h. Finally, the P3HT NPs dispersion was centrifuged at 500 rpm for 10 min to separate macroscopic aggregates.

### 4.2 Optical and Electrochemical Characterization

#### 4.2.1 UV–vis Absorption

Light absorption measurements were performed with a Perkin–Elmer Lambda 1050 spectrophotometer. The samples were prepared by placing the dispersions into disposable cuvettes with a dilution factor of 3/10 in ultrapure water.

#### 4.2.2 PL

The photoemission spectra were acquired using a HORIBA Jobin Yvon NanoLog spectrofluorometer. Sample preparation followed the same procedure as for the UV–vis measurements.

#### 4.2.3 Dynamic Light Scattering

The average nanoparticle size was evaluated through Dynamic Light Scattering (DLS) technique using a Malvern Zetasizer (Nano ZS, Malvern Instruments, U.K.). The laser wavelength was 633 nm (at an angle of 173° at 25°C), and measurements were performed in disposable polystyrene cuvettes, containing the dispersions diluted 1:10 in ultrapure water. The particle size distributions, for each batch, were obtained by averaging 10 independent measurements. The mean diameter and PDI values reported in the results section were the result of the average over all the prepared batches (n = 18).

#### 4.2.4 Photocurrent Measurements

Chronoamperometry measurements of 3P NPs colloidal dispersions were performed in a two‐electrodes configuration by employing a potentiostat/galvanostat (Autolab, PGSTAT 302N) and by applying constant bias equal to the Open Circuit Potential (OCP) value in dark conditions (0.12 V vs reference electrode). NPs dispersions were diluted in phosphate buffer solution (PBS 10 mM, pH 7) and were placed inside an electrochemical cell where an indium tin oxide (ITO) slab, in direct contact with the NPs, served as the working electrode (WE), and a platinum wire as the counter electrode (CE). Illumination was provided by a LED light source (emission peak, 530 nm, 38 mW/cm^2^).

### 4.3 HUVEC and H5V In Vitro Cell Cultures

#### 4.3.1 HUVECS

Primary Human Umbilical Vein Endothelial Cells (HUVECs), single donor, were purchased from Merck (Catalogue number, C‐12200), and cultured in Endothelial Cell Growth Medium 2 (Promocell) supplemented with the following Media Supplements (Promocell): Fetal Calf Serum (0.02 mL/mL), recombinant human Epidermal Growth Factor (5 ng/mL), recombinant human Basic Fibroblast Growth Factor (10 ng/mL), recombinant human Insulin‐like Growth Factor (Long R3 IGF, 20 ng/mL), recombinant human Vascular Endothelial Growth Factor 165 (0.5 ng/mL), Ascorbic Acid (1 µg/mL), Heparin (22.5 µg/mL), Hydrocortisone (0.2 µg/mL), and antibiotics (penicillin and streptomycin, 100 U/mL each). Cells were maintained in T‐75 culture flasks pre‐coated with a gelatin solution and incubated at 37°C with 5% CO_2_. Upon reaching 80%–90% confluence, cells were detached using 3% trypsin‐0.2% EDTA (Sigma Aldrich) for 5 min and seeded onto glass substrates for experiments. The glass coverslips were pre‐treated with fibronectin (from bovine plasma, 2 mg/ml in PBS, Sigma Aldrich) for 30 min to promote cell adhesion.

#### 4.3.2 H5V

Endothelial murine microvascular cardiac cells (H5V, RRID:CVCL_AZ87), kindly donated by Dr. A. Vecchi (Humanitas), were cultured in DMEM (Lonza) supplemented with 10% FBS (Lonza), 2 mM glutamine, 1 mM sodium pyruvate, and 100 U/ml penicillin‐streptomycin. Cells were incubated at 37°C with 5% CO_2_ in a humidified incubator. Upon reaching 80%–90% confluence, cells were detached using 0.5% trypsin‐0.2% EDTA (Sigma Aldrich) for 5 min and seeded onto glass substrates for experiments. The glass coverslips were pre‐treated with fibronectin (from bovine plasma, 2 mg/ml in PBS, Sigma Aldrich) for 30 min to promote cell adhesion.

### 4.4 AlamarBlue Proliferation Assay

HUVECs were seeded in 24‐well plates pre‐treated with 0.1% gelatin, at a density of 1×10^4^ cells/cm^2^. 3 h after plating, cells were treated with 3P NPs dispersed in MilliQ water (O.D. = 0.1, final dilution 1:10). After 6 h, excess non‐internalized NPs were removed by rinsing 3 times with cell medium, and photostimulation was performed for 6 h with a pulsed green light protocol (λ = 530 nm, 6 mW/cm^2^, 100 ms ON / 900 ms OFF). Optical excitation was provided by a LED multi‐array system (Teleopto LEDA‐G, provided by Green Leaf Technologies LTD), equipped with dedicated single channel LED driver LAD1 and triggered via TTL input by a pulse generator (STOmK‐2). The LED array geometry is compatible with standard, commercial multiwell plates, and it guarantees homogeneous illumination all over the cell substrate area, impinging from the top side (light spot size coincident with the well area), without overlapping with adjacent wells. The illumination system is waterproof and is compatible with the continuous use within the cell incubator, throughout the whole duration of the experiments. Moisture, which may change the photoexcitation density and homogeneity over time, was never observed. All photoexcitation experiments were carried out at fixed temperature of 37°C, the heating from the LED being completely negligible, as directly verified by an immersion thermocouple.

Cell proliferation was evaluated at 24, 48, and 168 h post‐treatment using the AlamarBlue assay (Thermo Fisher). For each time point, the culture medium was replaced with fresh medium supplemented with AlamarBlue at a final concentration of 100 mg/mL. Cells were incubated for 3 h at 37°C, 5% CO_2_, in the dark, with AlamarBlue. Resazurin, the active component of AlamarBlue, is reduced to resorufin in metabolically active cells, generating a fluorescent signal proportional to cell viability and proliferation. Following incubation, three 100‐µL aliquots of conditioned medium were transferred to a black 96‐well plate, and fluorescence was measured using a TECAN Spark microplate reader (excitation: 530 nm; emission: 590 nm). The assay was performed in three independent biological replicates.

### 4.5 Intracellular ·NO Detection

Intracellular ·NO was measured using the cell‐permeant probe DAF‐FM Diacetate (4‐Amino‐5‐Methylamino‐2',7'‐Difluorofluorescein Diacetate; Thermo Fisher). DAF‐FM diacetate (DA) is cell‐permeant and passively diffuses across cellular membranes. Upon cellular uptake, DAF‐FM‐DA is deacetylated by intracellular esterases to DAF‐FM, which reacts with nitrosating species to form a stable fluorescent triazole product (excitation/emission ≈ 495/515 nm). DAF‐FM is widely used for intracellular ·NO detection due to its relatively high specificity for ·NO under quasi‐physiological conditions. The fluorescence quantum yield of DAF‐FM is ∼0.005, but increases about 160‐fold, to ∼0.81, after reacting with NO [86]. As demonstrated by [87], the fluorescence increase of DAF‐FM is predominantly attributable to ·NO, while reactions with other RNS such as peroxynitrite (ONOO^−^) are of secondary importance under physiological redox conditions. While indirect reactions with strong oxidants cannot be entirely excluded, its fluorescence increase is considered a reliable proxy for changes in intracellular ·NO levels when appropriate controls are used.

HUVECs and H5V cells were seeded on bovine fibronectin–coated (1:50 dilution from stock concentration, Sigma‐Aldrich) glass coverslips in 24‐well plates at a density of 1.5×10^4^ cells/cm^2^. 3 h after plating, cells were incubated with 3P NPs dispersed in MilliQ water (O.D. = 0.1; final dilution 1:10). After 6 h, excess non‐internalized NPs were removed by rinsing, and photostimulation was applied according to the protocol reported in paragraph 5.4.

Measurements were performed 24 h after plating, by incubating cells with 10 µM DAF‐FM Diacetate in extracellular KRH solution, for 30 min at 37°C with 5% CO_2_ in the dark. After careful wash‐out of the excess probe from the extracellular medium with KRH extracellular solution, the fluorescence of the probe was recorded (photoexcitation density 1.5 mW/mm^2^ integration time 50 ms, 100 MHz, binning 1) with an upright microscope (Olympus BX63) equipped with a 20× water immersion objective, a spinning disk confocal module (X‐Light V2 spinning disk module from Crest Optics) and a sCMOS Camera (Prime BSI, Teledyne Photometrics; Tucson, Arizona, USA).

The fluorescence of the probe was recorded from multiple regions of the same sample that had not been previously exposed to photoexcitation. DAF‐FM was used as a relative indicator of intracellular ·NO‐related nitrosative activity, and fluorescence changes were compared exclusively between matched experimental conditions acquired using identical probe loading, illumination, and acquisition parameters, thereby minimizing systematic artifacts related to probe oxidation or background fluorescence. The variation of fluorescence intensity relative to the background was evaluated over Regions Of Interest (ROI) corresponding to single cells areas. Mean values were obtained by averaging measurements from multiple cells and fields of view per condition, across three independent biological replicates. All fluorescence measurements were performed under strictly identical acquisition settings, and data were analyzed as relative ·NO‐dependent fluorescence changes, normalized to the control dark condition.

Image processing was performed with ImageJ, and data were processed in Origin 2020. Data are represented as mean ± SEM. Statistical analysis was carried out using the Mann‐Whitney U‐test. *p*‐values: *** for *p* < 0.001, ** for *p* < 0.01, * for *p* < 0.05.

### 4.6 TRPL Measurements of NADH

HUVECs were seeded on bovine fibronectin–coated (1:50 dilution from stock concentration, Sigma‐Aldrich) glass coverslips in 24‐well plates at a density of 1.5×10^4^ cells/cm^2^. 3 h after plating, cells were incubated with 3P NPs dispersed in MilliQ water (O.D. = 0.1; final dilution 1:10). After 6 h, excess non‐internalized NPs were removed by rinsing three times with cell culture medium. Photostimulation, when applied, was performed the following day using the photoexcitation protocol described in paragraph 5.4. Time‐resolved photoluminescence (TRPL) measurements were then performed to monitor NADH photoluminescence (PL) dynamics in time up to 2.2 ns after laser excitation at 355 nm, enabling quantification of rapid metabolic responses. TRPL experiments were carried out using a femtosecond laser source coupled to a streak camera detection system (Hamamatsu C5680). A Ti:sapphire laser (Coherent Chameleon Ultra II, pulse bandwidths of B140 fs, repetition rate of 80 MHz, and maximum pulse energy of 50 nJ) was used to pump a second‐ harmonic crystal (b‐barium borate) to tune the pump wavelength to 355 nm. The sample has been mounted in a home‐made microscope with a 20× objective. The measurements here shown were performed recording the first 2 ns of decays, with an IRF of 8 ps.

Measurements were performed by using brightfield to find cells nuclei and focus the laser spot on the area where most mitochondria are concentrated. Laser spot has an estimated diameter of 2 µm, with which we excite NADH around mitochondria and record the PL from 450 nm to 500 nm. We move the spot around this area optimizing the area based on PL counts, finding the largest pool of NADH. Then, we record PL decay for 2 min and repeat this procedure for multiple cells.

The fluorescence decay *I*(*t*) of NADH can be described by a biexponential function [65, 88]:

$$I\;\left(t\right)=a_{1}\;exp\left(-\frac{t}{\tau_{1}}\right)+a_{2\;}exp\left(-\frac{t}{\tau_{2}}\right)$$

where τ_1_ and τ_2_ are the fluorescence lifetimes of the free and bound NADH, respectively, and *a*_1_ and *a*_2_ are the corresponding amplitude coefficients, reflecting the relative contributions of each species.

We define the fluorescence‐weighted fractional contributions as:

$$f_{1}=\frac{a_{1}\tau_{1}}{a_{1}\tau_{1}+a_{2}\tau_{2}}\;;f_{2}=\frac{a_{2}\tau_{2}}{a_{1}\tau_{1}+a_{2}\tau_{2}}\;$$

Here, *f*_1_ represents the fraction of the total fluorescence attributable to the free NADH component (τ_1_), typically elevated under glycolytic metabolic conditions. Conversely, *f*_2_ corresponds to the contribution of the total fluorescence associated with the component with decay time τ_2_: enzyme ‐bound NADH. A high *f*_2_ (more bound NADH) is typical of mitochondrial oxidative phosphorylation metabolism. We assume that the lifetimes τ_1_ and τ_2_ of both free and bound NADH in different cells are fixed, and remain invariant across samples of the same cell line. This assumption is grounded in their nature as quantum mechanical properties that depend primarily on the solvent environment (i.e., through solvatochromic effects), which is held constant across all measurements. Accordingly, during the fitting procedure, τ_1_ and τ_2_were held fixed while only the amplitude coefficients *a*_1_ and *a*_2_ were allowed to vary. This approach enables correlation of the relative contributions of each component to the concentrations of free and bound NADH. A custom MATLAB script was developed to implement this fitting methodology uniformly across all experimental samples.

### 4.7 Immunofluorescence Staining and Confocal Imaging

HUVECs were seeded on bovine fibronectin–coated (1:50 dilution from stock concentration, Sigma‐Aldrich) glass coverslips in 24‐well plates at a density of 2×10^4^ cells/cm^2^. 3 h after plating, cells were treated with 3P NPs dispersed in MilliQ water (O.D. = 0.1; final dilution 1:10). After 6 h, excess non‐internalized NPs were removed by rinsing. Photostimulation, when applied, was performed the following day using the photoexcitation protocol described in paragraph 5.4. To preserve light‐induced effects, immediately after light stimulation, cells were washed with PBS and fixed with Antigenfix solution (Diapath). For the evaluation of iNOS and eNOS expression, cells were stained with Orange CellMask Plasma Membrane Stain (Thermo Fisher; 1:1000 dilution in PBS, 7 min at 37°C) prior to fixation. Following fixation, cells were permeabilized with 0.1% Triton X‐100 (Sigma Aldrich) in 1% PBS for 10 min at room temperature, then washed twice with PBS. Blocking was performed with 5% bovine serum albumin (BSA; Sigma Aldrich) in 1% PBS for 1 h at room temperature. After two PBS washes, cells were incubated overnight at 4°C with either: (i) primary mouse anti‐CD31 antibody (BioLegend, catalogue no. 102401) for membrane staining in internalization experiments, or (ii) primary rabbit anti‐eNOS or anti‐iNOS antibodies (Thermo Fisher Scientific, catalogue no. PA5‐16887 and PA1‐036, respectively), diluted 1:500, 1:250, and 1:500, respectively, in 1% BSA in PBS. Samples were washed twice with PBS containing 0.05% Tween‐80 (PBST) to remove unbound antibodies. Secondary antibody incubation was performed for 2h at room temperature, using FAB2 anti‐mouse IgG red‐fluorescent secondary antibody (Thermo Fisher Scientific, catalogue no. A‐21053) for CD31 staining, or Alexa Fluor 488 goat anti‐rabbit IgG (H+L) cross‐adsorbed secondary antibody (Thermo Fisher Scientific, catalogue no. A‐11008; 1:500 dilution in 1% BSA in PBS) for eNOS/iNOS staining. Cells were washed three times with PBST (5 min per wash). For internalization experiments, actin filaments were stained with phalloidin‐FITC (Sigma‐Aldrich; 1:1000 in 1% BSA in PBS, 5 min), followed by three PBST washes. Nuclei were counterstained with Hoechst 33342 (Thermo Fisher; 1 µg/mL in PBS) for 10 min at room temperature in the dark, followed by two PBS washes. Samples were stored in PBS at 4°C until imaging.

Confocal images were acquired using inverted confocal laser scanning microscope Nikon Eclipse Ti2 (Nikon Instruments), equipped with a 60× oil objective, 403, 487, 561, and 636 nm laser sources and 450/50, 525/50, 595/50, and 700/75 nm emission filter sets. The excitation/emission conditions of the employed probes are depicted in Table 1.

**TABLE 1:** excitation/emission acquisition conditions of the employed fluorescent probes.

Fluorescent probe	Laser source (nm)	Emission filter (nm)
Hoechst 33342	403	450/50
CellMask Orange	561	595/50
Phalloidin‐FITC	487	525/50
FAB2	638	700/75
Alexa Fluor 488	487	525/50

For eNOS and iNOS fluorescence analysis, 15< N < 20 fields were measured for each condition (each field containing at least 15 cells) across three experimental replicates and two biological replicates. Image analysis was performed on z‐stack laser scanning images using Fiji (ImageJ). First, a sum intensity z‐projection was generated. CellMask channel was used to create a mask by applying the default ImageJ threshold to identify cells and generate regions of interest (ROIs). This mask was applied to the eNOS/iNOS channels to extract the mean fluorescence intensity within the cells. A background ROI was also selected on the eNOS/iNOS channels to measure and subtract the mean background intensity. Finally, data from each experimental day were normalized to the average intensity of the dark control group.

## Conflicts of Interest

The authors declare no conflict of interest.

## Supporting information




**Supporting File**: advs74448‐sup‐0001‐SuppMat.pdf.

## Data Availability

The data that support the findings of this study are available from the corresponding author upon reasonable request.
