# Supplementary material for: Semiconducting Polymer Nanoparticles Enable Light‐Controlled Bidirectional Modulation of Nitric Oxide in Endothelial Cells
Source: Adv Sci (Weinh). 2026 Feb 20;13(30):e22894. doi: 10.1002/advs.202522894 (PMC13248851; doi:10.1002/advs.202522894)
Supplement: Supplementary file 1 — Supporting File: advs74448‐sup‐0001‐SuppMat.pdf. [file ADVS-13-e22894-s001.pdf]

Semiconducting Polymer Nanoparticles Enable Light-Controlled Bidirectional Modulation of Nitric Oxide in Endothelial Cells

Camilla Marzuoli[1], Andrea Pianetti[1], Elena Mancinelli[1], Anthea Villano[1], Matteo Vailati[1], Paola Lagonegro[1], Miryam Criado-Gonzalez[2], Montserrat Climent-Salarich[3,4], Hansel Comas-Rojas[1], Leonardo Elia[4,5], Francesco Moccia[6], Gabriele Tullii[1,*], Maria Rosa Antognazza[1,*]

[1] Center for Nano Science and Technology, Istituto Italiano di Tecnologia, Via Raffaele Rubattino 81, 20134 Milano, Italy

[2] Institute of Polymer Science and Technology (ICTP-CSIC), Juan de la Cierva 3, 28006 Madrid, Spain

[3] Department of Biomedical Sciences, Humanitas University, 20072 Pieve Emanuele, Italy

[4] Humanitas Cardio Center, IRCCS Humanitas Research Hospital, 20089 Rozzano, Italy

[5] Department of molecular and translational medicine, University of Brescia, Brescia, Italy

[6] Department of Medicine and Health Sciences "V. Tiberio", University of Molise, Via Giovanni Paolo II, 86100 Campobasso, Italy

*gabriele.tullii@iit.it ; mariarosa.antognazza@iit.it

Supporting Information

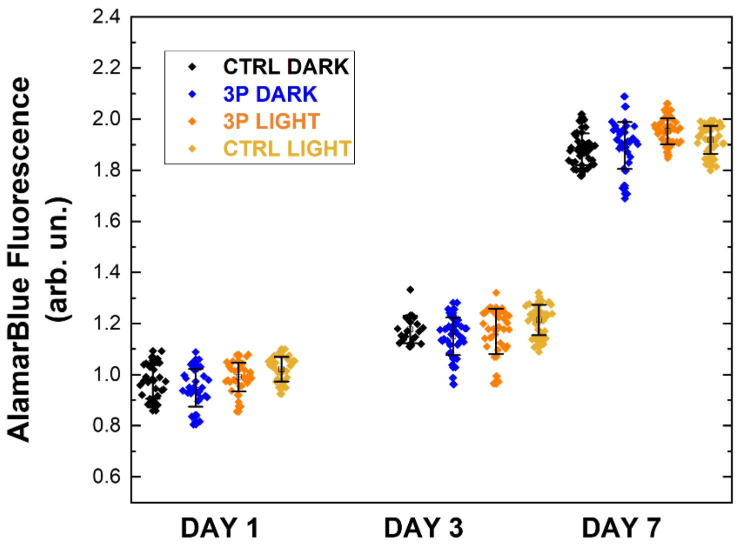

Figure S1: AlamarBlue proliferation assay of HUVECs treated with 3P NPs evaluated as the fluorescence of the reduced form of the AlamarBlue cell-viability reagent. NPs (O.D. = 0.1) were added to cell culture 3 h after plating, while light treatment (λ = 530 nm, 6 $\mathbf{mW/cm^2}$, 100 ms ON / 900 ms OFF, for 6 h) was performed the following day, prior to measurements. Data have been normalized to control sample, i.e., control dark at 24 h. Error bars represent the standard deviation (SD).

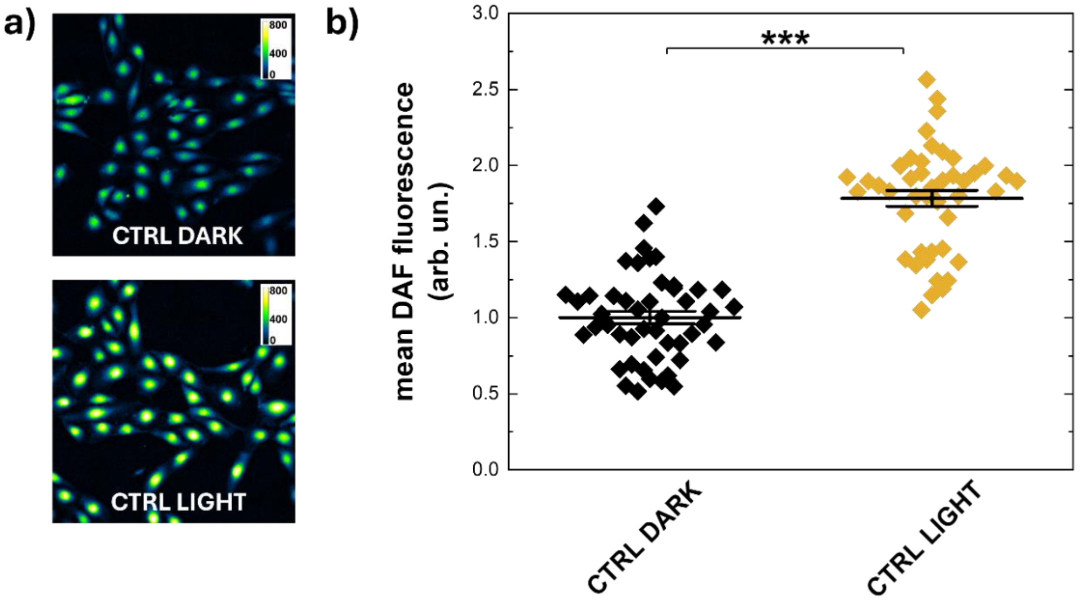

Figure S2: Intracellular ·NO concentration in HUVECs, evaluated by the measurement of the NO-sensitive fluorophore DAF fluorescence intensity. a) Images depicting representative fields for controls. b) Data representing the mean DAF fluorescence for each condition. Each dot represents a field of view, with each field averaging at least 15 cells; total number of cells $650 < n < 700$ for each condition. Light treatment ($\lambda = 530$ nm, 6mW/cm^2, 100 ms ON / 900 ms OFF, for 6 h) was performed the day after plating, prior to measurements. Data are represented as mean ± SEM values, normalized to control dark. Anova test 2-ways, Bonferroni correction. P-values: *** for $p < 0.001$.

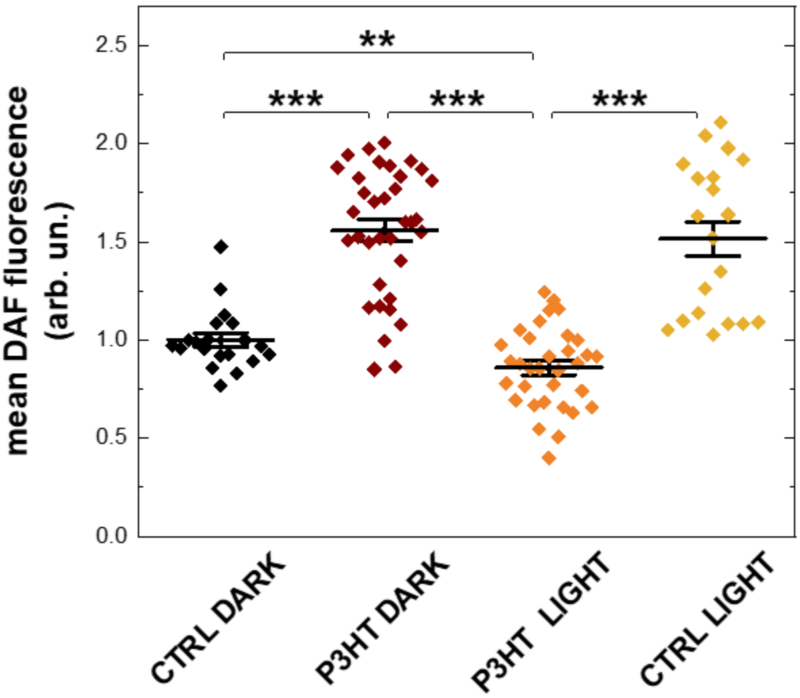

Figure S3: Intracellular ·NO concentration in HUVECs, evaluated by the measurement of the ·NO -sensitive fluorophore DAF fluorescence intensity. P3HT NPs 20 ug/ml were added to cell culture 3 h after plating, while light treatment (λ = 530 nm, 6mW/cm^2, 100 ms ON / 900 ms OFF, for 6 h) was performed the day after plating, prior to measurements. Each dot represents a field of view, with each field averaging at least 15 cells. Data are represented as mean ± SEM, normalized to control dark. Statistical analysis was performed on three independent biological replicates using the Anova test 2-ways, Bonferroni correction. Significance values:*** for $p < 0.001$; ** for $p < 0.01$; * for $p < 0.05$.

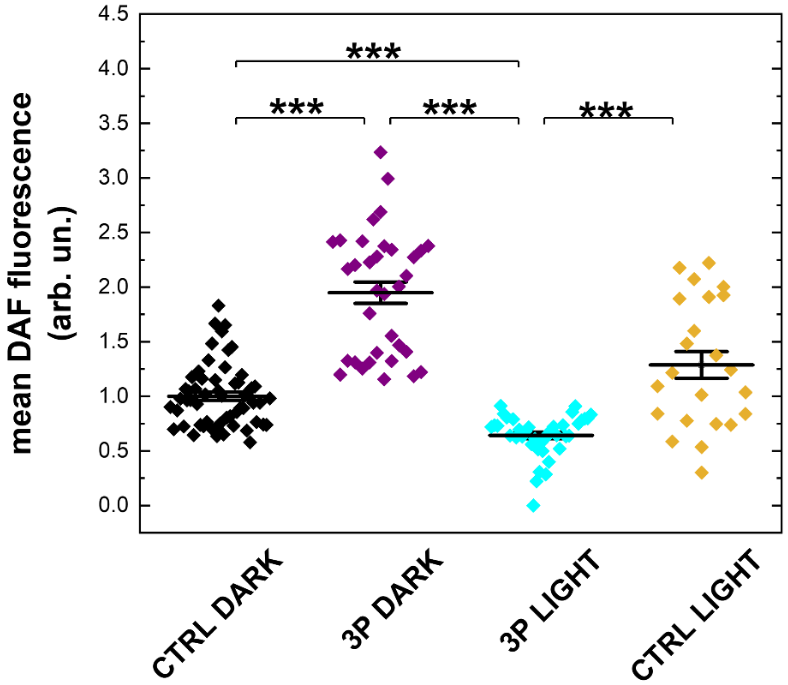

Figure S4: Intracellular ·NO concentration in H5V cells, evaluated by the measurement of the ·NO -sensitive fluorophore DAF fluorescence intensity. 3P NPs were added to cell culture 3 h after plating, while light treatment ($\lambda = 530$ nm, 0.6 mW/cm^2, 100 ms ON / 900 ms OFF, for 6 h) was performed the day after plating, prior to measurements. Data are represented as mean ± SEM values, normalized to control dark. Each dot represents a field of view, with each field averaging at least 15 cells. Data are represented as mean ± SEM, normalized to control dark. Statistical analysis was performed on three independent biological replicates using the Anova test 2-ways, Bonferroni correction. Significance values: *** for $p < 0.001$; ** for $p < 0.01$; * for $p < 0.05$.

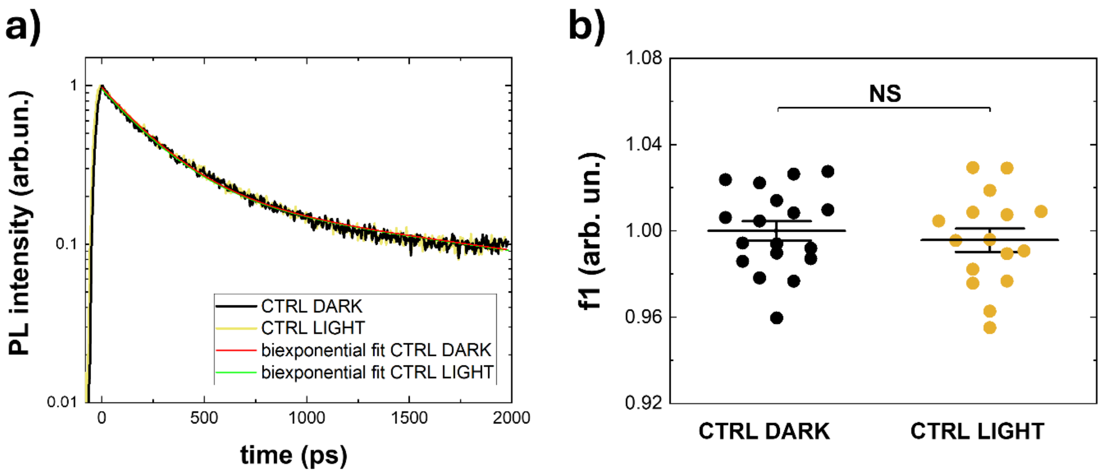

Figure S5: TRPL analysis of NADH metabolism in untreated HUVECs (CTRL DARK) and HUVECs after photostimulation (CTRL LIGHT). Light treatment ($\lambda = 530$ nm, 6 mW/cm^2, 100 ms ON / 900 ms OFF, for 6 h) was performed the day after plating, prior to measurements. a) Representative decay curves fitted using a biexponential decay model to extract the average lifetimes of free and enzyme -bound NADH. b) Quantification of the fractional NADH free component $\boldsymbol{f_1}$, as derived from the biexponential fit. Each data point corresponds to an individual cell. Values are represented as mean ± SEM, normalized to the control dark condition, and averaged over three biological replicates. Statistical analysis was conducted using the Mann-Whitney U-test on three independent biological replicates. NS = not significant.

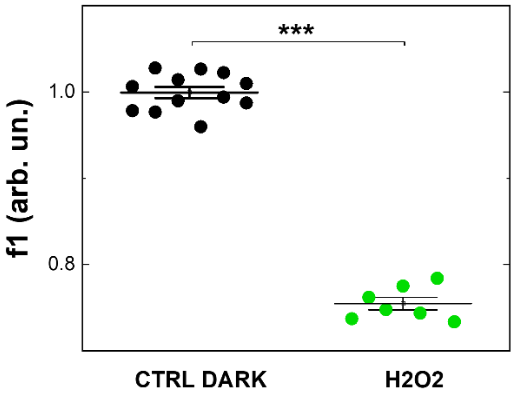

Figure S6: TRPL analysis of NADH metabolism in untreated HUVECs (CTRL DARK) and $\mathbf{H_2O_2}$ - treated cells ($\mathbf{H_2O_2}$). Cells were treated with 1 **μM** exogenous $\mathbf{H_2O_2}$ for 30 min prior to measurement. Quantification of the fractional contribution of free NADH ($\boldsymbol{f_1}$) is derived from the biexponential fit across different conditions. Each data point corresponds to an individual cell. Values are represented as mean ± SEM, normalized to control dark. Statistical significance determined by Mann-Whitney U-test. Significance level: *** for $p < 0.001$.

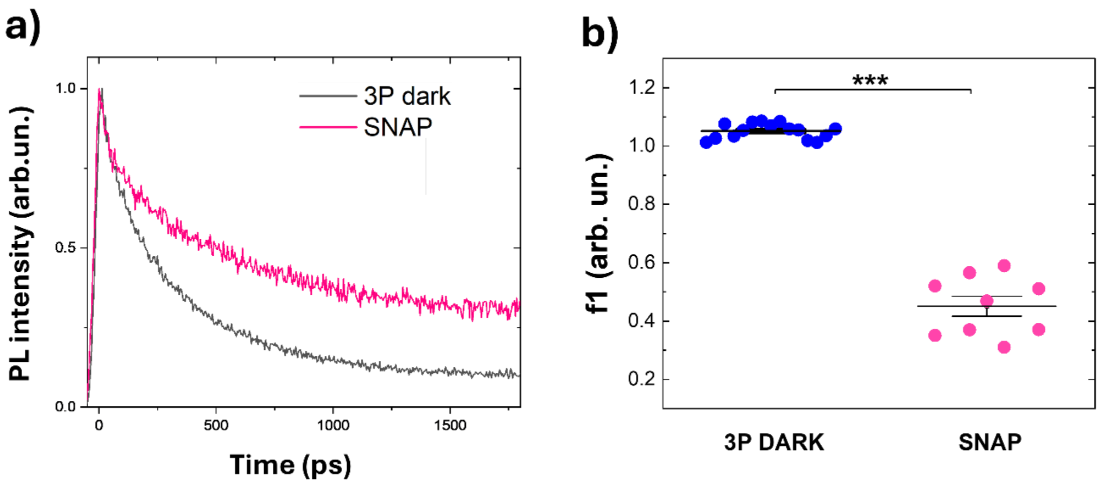

Figure S7: TRPL analysis of NADH metabolism in HUVECs. a) Representative NADH fluorescence decay curves of 3P-treated HUVECs and SNAP-treated HUVECs. 3P NPs were added to cell culture 3 h after plating, while SNAP treatment (200 **μM**) was added to the cell culture 30, 45 and 60 min before measurements respectively. b) Quantification of the fractional contribution of free NADH ($\boldsymbol{f_1}$) derived from the biexponential fit across different conditions. Each data point corresponds to an individual cell. Values are represented as mean ± SEM, normalized to control dark. Statistical significance determined by Mann-Whitney U-test. Significance level: *** for $p < 0.001$.

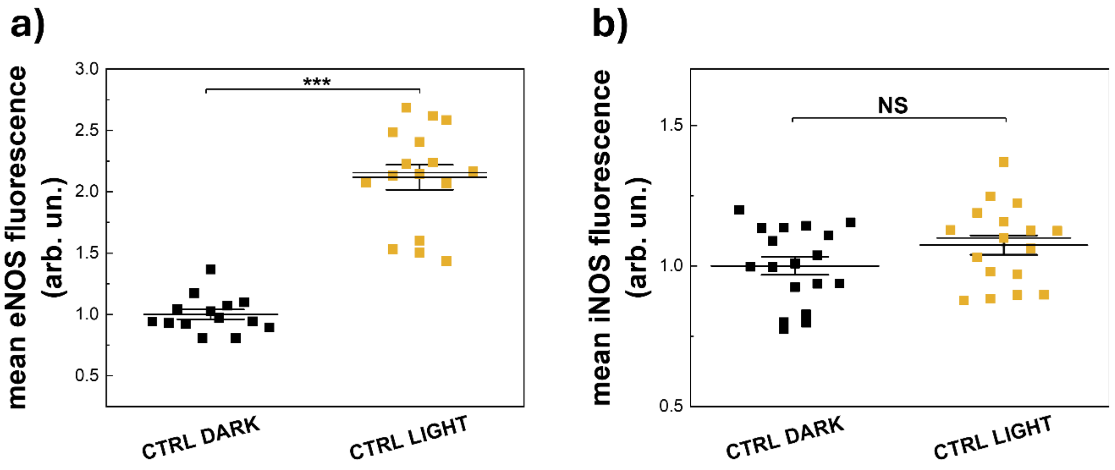

Figure S8: a) eNOS and b) iNOS expression in HUVECs determined via immunostaining. Each data point represents a field of view, with each field averaging at least 50 cells. Light treatment (λ = 530 nm, 6 mW/cm^2, 100 ms ON / 900 ms OFF, for 6 h) was performed the day after plating, prior to measurements. Data are represented as mean ± SEM values, normalized to control dark. Statistical analysis: one-way ANOVA with Tukey's post hoc test. Significance levels: ***$p < 0.001$.
